# Examining longitudinal associations between prenatal exposure to infections and child brain morphology

**DOI:** 10.1016/j.bbi.2024.05.014

**Published:** 2024-05-13

**Authors:** Anna Suleri, Carolin Gaiser, Charlotte A.M. Cecil, Annet Dijkzeul, Alexander Neumann, Jeremy A. Labrecque, Tonya White, Veerle Bergink, Ryan L. Muetzel

**Affiliations:** a Department of Child and Adolescent Psychiatry/Psychology, Erasmus University Medical Center, the Netherlands; b The Generation R Study Group, Erasmus University Medical Center, Rotterdam, the Netherlands; c Department of Neuroscience, Erasmus University Medical Center, Rotterdam, the Netherlands; d Department of Epidemiology, Erasmus University Medical Center, Rotterdam, the Netherlands; e Department of Biomedical Data Sciences, Molecular Epidemiology, Leiden University Medical Center, Leiden, the Netherlands; f Section on Social and Cognitive Developmental Neuroscience, National Institute of Mental Health, Bethesda, MD, USA; g Department of Psychiatry, Icahn School of Medicine at Mount Sinai, NY, USA; h Department of Psychiatry, Erasmus University Medical Center, Rotterdam, the Netherlands; i Department of Radiology and Nuclear Medicine, Erasmus University Medical Center, Rotterdam, the Netherlands

**Keywords:** Prenatal maternal infection, Maternal immune activation, Neurodevelopment, Pediatric neuroimaging, Population-based cohort

## Abstract

**Background:**

Maternal infection during pregnancy has been identified as a prenatal risk factor for the later development of psychopathology in exposed offspring. Neuroimaging data collected during childhood has suggested a link between prenatal exposure to maternal infection and child brain structure and function, potentially offering a neurobiological explanation for the emergence of psychopathology. Additionally, preclinical studies utilizing repeated measures of neuroimaging data suggest that effects of prenatal maternal infection on the offspring’s brain may normalize over time (i.e., catch-up growth). However, it remains unclear whether exposure to prenatal maternal infection in humans is related to long-term differential neurodevelopmental trajectories. Hence, this study aimed to investigate the association between prenatal exposure to infections on child brain development over time using repeated measures MRI data.

**Methods:**

We leveraged data from a population-based cohort, Generation R, in which we examined prospectively assessed self-reported infections at each trimester of pregnancy (N = 2,155). We further used three neuroimaging assessments (at mean ages 8, 10 and 14) to obtain cortical and subcortical measures of the offspring’s brain morphology with MRI. Hereafter, we applied linear mixed-effects models, adjusting for several confounding factors, to estimate the association of prenatal maternal infection with child brain development over time.

**Results:**

We found that prenatal exposure to infection in the third trimester was associated with a slower decrease in volumes of the pars orbitalis, rostral anterior cingulate and superior frontal gyrus, and a faster increase in the middle temporal gyrus. In the temporal pole we observed a divergent pattern, specifically showing an increase in volume in offspring exposed to more infections compared to a decrease in volume in offspring exposed to fewer infections. We further observed associations in other frontal and temporal lobe structures after exposure to infections in any trimester, though these did not survive multiple testing correction.

**Conclusions:**

Our results suggest that prenatal exposure to infections in the third trimester may be associated with slower age-related growth in the regions: pars orbitalis, rostral anterior cingulate and superior frontal gyrus, and faster age-related growth in the middle temporal gyrus across childhood, suggesting a potential sensitive period. Our results might be interpreted as an extension of longitudinal findings from preclinical studies, indicating that children exposed to prenatal infections could exhibit catch-up growth. However, given the lack of differences in brain volume between various infection groups at baseline, there may instead be either a longitudinal deviation or a subtle temporal deviation. Subsequent well-powered studies that extend into the period of full brain development (~25 years) are needed to confirm whether the observed phenomenon is indeed catch-up growth, a longitudinal deviation, or a subtle temporal deviation.

## 1 Introduction

The prenatal period signifies an important period in which the brain undergoes substantial development during each trimester (neuron production in early pregnancy versus synaptogenesis and myelination in late pregnancy) (O’Rahilly and Müller, 2008; Prayer et al., 2006). Exposure to infections in utero has been proposed to be one of the environmental factors that may impact fetal neurodevelopment (Han et al., 2021; Han et al., 2021). There are several hypothesized pathways through which prenatal infections may affect the offspring’s brain (Han et al., 2021; Estes and McAllister, 2016). Specifically, infectious pathogens may directly pass the placenta and assert their effect (e.g., severe infections such as HIV, Herpes, Rubella, or Cytomegalovirus), or rather than pathogen specific effects, they may lead to the activation of the mother’s immune system due to systemic inflammation, after which the immune cells on the placenta may consequently be activated (Han et al., 2021). As a result, inflammatory markers can be increased in the fetal circulation which could then impact the regulatory role of immune cells in the brain (such as microglia) and consequently interrupt typical fetal neurodevelopment due to neuroinflammation (Han et al., 2021). Moreover, placental immune activation may lead to epigenetic modifications, which in turn may lead to neuroinflammation by impacting microglia due to gene regulation (Han et al., 2021). Indeed, numerous systematic reviews have demonstrated relationships between prenatal maternal infections and a wide array of mental health disorders in offspring, such as depression and schizophrenia (al-Haddad et al., 2019; Fung et al., 2022; Khandaker et al., 2013). However, our understanding of the potential biological mechanisms underlying this association remains limited. As a result, there has been growing interest in studying the brain to shed light on potential biological mechanisms (Marek et al., 2022).

A wealth of animal studies has demonstrated both short- and long-term effects of prenatal exposure to infections on the offspring’s brain, as evidenced by a recent systematic review (Guma et al., 2019). In humans, a small set of studies have reported morphological differences in certain brain regions (e.g., in the temporal, parietal, and frontal lobe) during infancy (Birnbaum et al., 2017; Lipitz et al., 2010) after prenatal exposure to infections. Moreover, in a previous study from our group using data from the general population, we found that prenatal exposure to infections associated with a smaller caudal anterior cingulate volume in adolescence (age 14 years), as well as nominal associations with smaller volume of frontotemporal regions, although most regions showed no statistically significant association (Suleri et al., 2023). Prior studies in humans, using inflammatory biomarkers to define maternal immune activation, have found brain and behavioral alterations in newborns and toddlers (e.g., in the hippocampus, amygdala and white matter) (Graham et al., 2018; Rudolph et al., 2018; Rasmussen et al., 2022; Rasmussen et al., 2019) and a reduction in cerebellar volume in pre-adolescents (Suleri et al., 2022). Interestingly, studies in animals with repeated measures of neuroimaging data have demonstrated an age-dependent association between prenatal infection and child brain development (Crum et al., 2017; Mueller et al., 2021; Guma et al., 2021). Specifically, at an early age, reductions in brain volume were shown in mice offspring exposed to prenatal infections, yet these brain volume differences tended to normalize by adulthood, where exposed mice eventually showed similar brain volumes as unexposed mice (i.e., showing catch-up growth) (Crum et al., 2017). This pattern was observed in mice offspring exposed to both normal intensity as well as high-intensity prenatal infection models (Richetto et al., 2017). The notion of catch-up growth suggests a compensatory mechanism wherein the brain strives to mitigate potential deficits that occurred during critical developmental periods. Elucidating this phenomenon may shed light on improving our understanding of the brain’s capacity for resilience.

Despite various single timepoint studies in early or late childhood, little is known about the longitudinal effects of prenatal infection on the developing brain in humans postnatally. As a result, it remains unclear how prenatal infection affects child brain development over time (Guma et al., 2019). Addressing this gap would enables us to characterize trajectories and differentiate between typical and atypical development (Di Martino et al., 2014). It can provide further insight into potential sensitive periods of the effect of prenatal infection on the offspring’s brain. This is especially important, given that early childhood to adolescence marks a dynamic and critical period for the complex development of the human brain, as evidenced by longitudinal magnetic resonance imaging (MRI) studies of typically developing individuals (Shaw et al., 2008; Vijayakumar et al., 2016; Sussman et al., 2016; Mills et al., 2016). For example, the prefrontal cortex, a region responsible for executive functions such as decision-making and impulse control, undergoes remarkable development during adolescence, even continuing into early adulthood (Arain et al., 2013). An additional benefit of repeated measures data is that it provides an elegant way to adjust for time-invariant individual differences (Mills and Tamnes, 2014). Moreover, the timing of infection during pregnancy may be an important factor to consider. The developing fetal brain undergoes a series of intricate and precisely timed processes, making certain gestational periods particularly vulnerable to external influences (Arain et al., 2013). Currently, this temporal dimension remains relatively understudied in both animal and human research. Exploring specific trimesters during which maternal infections exert the most significant impact could provide valuable insights into the timing-dependent vulnerabilities of the developing offspring’s brain.

Accordingly, the aim of this large population-based study was to investigate the longitudinal association between prenatal infection and child brain morphology development from early childhood to adolescence. We used a composite score to define maternal infections at each trimester in pregnancy. We further included three repeated measured of child brain morphology at child mean ages 8, 10 and 14 years.

## 2 Methods

### 2.1 Study selection and participants

Our study was embedded in the Generation R Study, an ongoing prospective cohort study designed to investigate the progression of child health, from fetal life to adulthood (Kooijman et al., 2016). The recruitment strategy has been explained previously (Jaddoe et al., 2007). Briefly, pregnant individuals from Rotterdam with an expected due date falling between April 2002 and January 2006 were invited to participate. Approximately 80 % of the mothers were included in the first trimester, 18 % of the mothers were included in the second trimester and 2 % of the mothers were included in the third trimester. After pregnancy, three subsequent waves have taken place. During the MRI research visits at child’s mean age of 8, mean age of 10, and mean age of 14 years, a total of 1,070, 3,992 and 3,725 children, respectively, took part (Figures S1-S2). The Generation R Study received approval from the Medical Ethics Committee at the Erasmus MC and was conducted in compliance with the principles outlined in the Declaration of Helsinki. Written informed consent was obtained from the parents, and when applicable, assent was obtained from their children.

To be included in the study, the following criteria had to be met: 1) complete information on infection exposure during pregnancy had to be available for each trimester (hence, to ensure the reliability of the trimester specific reports, only mothers enrolled in the first trimester were included) and 2), at least one of the three brain scans needed to be available. MRI scans that exhibited poor quality, significant incidental findings, or involved participants with dental braces were excluded from the study. Furthermore, in cases where study participants had siblings or twins, the sibling with the highest data availability was selected for inclusion. If siblings or twins had equal data availability, one sibling was randomly chosen to be included.

### 2.2 Assessment of prenatal infection

**Fig. 1:**
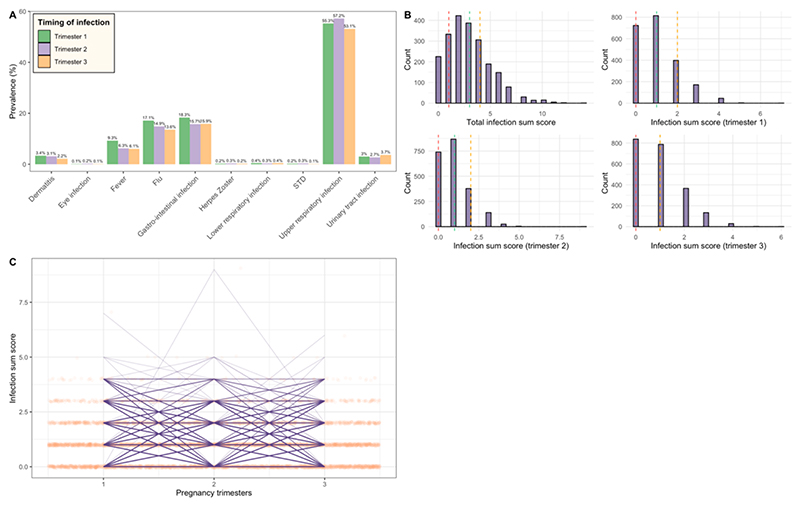
Fig. 1A depicts the prevalence per infection type per trimester Fig. 1B depicts the distribution of the infection sum score across trimesters, and Fig. 1C depicts the rate of infection per mother across gestation (including datapoints; and the thickness of the line indicates the number of women that had a similar infection rate). In Fig. 1B, the dotted line indicates the quartiles. Of note, Fig. 1B-trimester 3 only shows two lines as the median and third quartile have the same infection score (the second dotted line depicts the median).

Prenatal exposure to infections were measured using a previously constructed sum score containing multiple domains of infection types (Suleri et al., 2023). The infection domains were chosen based on their likelihood to cause systemic inflammation (Han et al., 2021; Saarentaus et al., 2023; Clementi et al., 2021; Bauer et al., 2023; de Jong et al., 2016; Blomqvist and Engblom, 2018; Mwatelah et al., 2019; den Heijer et al., 2019; Itamura and Sawada, 2022; Elgormus et al., 2023; Hannan et al., 2010; Miller and Hanumunthadu, 2022; McCluskey and Powell, 2004). This score was calculated using questionnaire data collected prospectively during each trimester of pregnancy. Pregnant individuals were asked about the occurrence of various types of infections shortly after each trimester. To ensure the reliability of the trimester specific reports, we only included pregnant women that were enrolled in the first trimester and who had complete data for the three questionnaires. Infection types that were asked about: 1) upper respiratory infections (such as pharyngitis, rhinitis, sinusitis, and ear infections), 2) lower respiratory infections (such as pneumonia and bronchitis), 3) gastrointestinal infections (such as diarrhea and enteritis), 4) cystitis/pyelitis, 5) dermatitis (such as boils and erysipelas), 6) eye infections, 7) herpes zoster, 8) sexually transmitted diseases, 9) flu, and/or 10) a period of fever (>38 °C/100.4°F). To reflect the occurrence of these specific conditions throughout pregnancy, we developed four summary scores: one for each trimester and one encompassing the entire duration of pregnancy. In this scoring system, the presence of each infection type was assigned one point, while the absence of an infection type was assigned zero points (i.e., max 30 points for the entire pregnancy and max 10 points per trimester). Each questionnaire at each time point was treated separately, and we did not have information if the infection was new or a continuation of an existing infection. The distribution of each infection type per trimester, as well as the distributions and the rate of the infection sum score can be found in Fig. 1.

### 2.3 Assessment of child brain morphology

We studied repeated measurements of brain morphology at child mean age 8 (neuroimaging visit 1), 10 (neuroimaging visit 2) and 14 years (neuroimaging visit 3) in which we investigated both cortical and subcortical regions.

#### 2.3.1 Image acquisition

We used T_1_-weighted structural MRI images to obtain measures of brain morphology. All children at mean age 8, 10 and 14 years were offered to participate in a mock MRI scanning session to become familiar with the MRI procedure (White et al., 2018). For neuroimaging visit 1 (child mean age 8), MRI images were collected using a 3-Tesla General Electric Discovery MR750 system, (GE, Milwaukee, WI) (White et al., 2013). For neuroimaging visits 2–3 (child mean age 10 and 14), a 3-Tesla GE Discovery MR750w (GE, Milwaukee, WI) system was used (Dall’Aglio et al., 2023). Both systems used an 8-channel receiving head coil. At neuroimaging visit 1 (child mean age 8), high resolution T_1_-weighted scans were obtained using an inversion recovery fast spoiled gradient recalled (IR-FSPGR) sequence with the following parameters: repetition time = 10.3 ms, echo time = 4.2 ms, inversion time = 350 ms, number of excitations = 1, flip angle = 16 degrees, matrix size = 256 x 256, ASSET imaging acceleration factor = 2, and the isotropic resolution = 0.9 mm^3^. At neuroimaging visits 2–3 (child mean age 10 and 14), high resolution T_1_-weighted scans were obtained with an IR-FSPGR sequence with the following parameters: repetition time = 8.77 ms, echo time = 3.4 ms, inversion-time = 600 ms, number of excitations = 1, flip angle = 10°, matrix size = 220 x 220, ARC imaging acceleration factor = 2, and the isotropic resolution = 1.0 mm^3^.

#### 2.3.2 Image processing

At all timepoints, the FreeSurfer analysis suite (version 6.0.0) [https://surfer.nmr.mgh.harvard.edu/] was used to process the data. All detailed steps have been previously outlined (Fischl, 2012). In summary, raw DICOM data was converted to ‘MGZ-files’, after which the images were stripped of the skull, normalized for the intensity (voxel intensities were corrected for B_1_ inhomogeneities), and segmented by voxels (to parcellate anatomic regions into gray and white matter and cerebrospinal fluid).

We included cortical regional volumes from the Desikan-Killiany atlas (Desikan et al., 2006). This atlas parcellates the brain into the following regions per lobe: *frontal lobe* (superior frontal, rostral and caudal middle frontal, pars opercularis, pars triangularis and pars orbitalis, lateral and medial orbitofrontal, precentral, paracentral and frontal pole), *parietal lobe* (superior parietal, inferior parietal, supramarginal, postcentral and precuneus), *temporal lobe* (superior, middle and inferior temporal, banks of the superior temporal sulcus, fusiform, transverse temporal, entorhinal, temporal pole, parahippocampal), *occipital lobe* (lateral occipital, lingual, cuneus, and pericalcarine) and *cingulate* (rostral anterior, caudal anterior, posterior, and isthmus). We further analyzed the cerebellum and several FreeSurfer-derived subcortical volumes, specifically, the amygdala, hippocampus, caudate, putamen, thalamus, and pallidum. Because we had no hypothesis on potential lateralized effects, we computed the average for each brain volume across both hemispheres. A correlation matrix of all exposure and outcome variables can be found in Figure S3.

#### 2.3.3 Image quality

To ensure the accuracy of the scans, a trained evaluator performed a visual examination of the scans. This review process included analyzing the boundaries between gray and white matter as identified by Free-Surfer. Each scan was assessed using a Likert scale, categorizing them as poor, questionable, or good. Any scans that were considered unusable or of low quality were excluded from the analysis. More information can be found in Supplemental Text A. Detailed information about this process has been previously provided (Muetzel et al., 2019; Steenkamp et al., 2022).

### 2.4 Inverse-probability weighting predictors and covariates

Several factors were included in statistical analyses, either as covariates in the linear mixed-effects models, or for the inverse-probability weighting procedure (See Section 2.5 Statistical Analyses below). Hospital registries were utilized to gather information about the sex assigned at birth. During enrollment, various details were collected through self-reported questionnaires, including maternal age, maternal national background (classified as ‘Dutch’ or ‘non-Dutch’), household income (categorized as ‘<€2,200/month’ or ‘>€2,200/month)’, maternal education (categorized as ‘primary’ [no education or primary school], ‘intermediate’ [secondary school or lower vocational training| and ‘high’ [higher vocational training or university]), maternal tobacco use (categorized as ‘no’, ‘yes, until pregnancy was known,’ and ‘yes, continued during pregnancy’), and prenatal selective serotonin reuptake inhibitor (SSRI) usage. Maternal psychopathology during pregnancy (at 20 weeks of gestation) was evaluated using the Brief Symptom Inventory which consisted of 53 items (de Beurs, 2004). From the responses, a Global Severity Index was computed, providing a quantitative assessment of maternal psychopathology on a continuous scale. A higher score on this index indicates a greater degree of problems. Age of participants at each MRI assessment was further used as a covariate. Additionally, intracranial volume (ICV) was measured with the MRI scan at each imaging wave. When the child was six years old, total behavioral problems score and autistic traits were measured with the Child Behavioral Checklist (CBCL) and Social Responsiveness Scale (SRS), respectively.

### 2.5 Statistical analyses

To answer our primary research question, we applied linear mixed-effects model (*lme4* and *broom.mixed* package). This model allowed us to examine the association between prenatal infection and brain morphology over time. We were first interested if the association between prenatal infection and brain morphology was stable or age related (i.e., is prenatal infection related to changes in brain morphology). To investigate this research question, we examined the interaction between total prenatal infection and time (defined as time of neuroimaging visit, i.e., at child mean age 8, at child mean age 10, and at child mean age 14) (marked bold in Equation 1). A random intercept was added to account for the repeated measurements per individual. The interaction term can be interpreted as the association between prenatal infection and change in brain morphology over time. As secondary analyses, to investigate whether there were trimester-specific associations, we repeated all analyses with trimester-specific infection sum scores instead of the total pregnancy infection sum score in separate models.

$$\begin{array}{l} {\text{Y}}_{\text{ij}}\;\text{\textasciitilde{}}\;\text{prenatal}\;{\text{infection}}_{i}{\text{:time}}_{\text{ij}}\;\text{+}\;\text{prenatal}\;{\text{infection}}_{i}\text{+}\;{\text{time}}_{\text{ij}}\;\text{+}\;\text{child}\\ {\text{sex}}_{i}\text{+}\;\text{maternal}\;{\text{age}}_{i}\text{+}\;\text{maternal}\;\text{national}\;{\text{background}}_{i}\;\text{+}\;\text{household}\\ {\text{income}}_{i}\text{+}\;\text{maternal}\;{\text{education}}_{i}\text{+}\;\text{maternal}\;\text{tobacco}\;{\text{use}}_{i}\;\text{+}\;\text{maternal}\\ \text{prenatal}\;{\text{psychopathology}}_{i}\text{+}\;{\mu 0}_{i}\text{+}\;{\text{ε}}_{\mathrm{ij}} \end{array}$$

Equation 1. Linear mixed-effects model. The *i* subscript indicates the participant, the *j* subscript indicates the timepoint. The term ‘Yij’ indicates the individual child brain morphology outcomes. The ‘prenatal infection_i_ * time_ij_’ is the term of interest of which the coefficient shows the association between prenatal infection and change in child brain morphology over time. Time is defined as time of MRI scan. The term μ0_i_ indicates the random intercept. Prenatal infection is entered as a continuous variable in this model. In this model we model both the main effect as well as the interaction effect; but only the interaction effect is interpreted.

We further conducted several sensitivity analyses. First, we repeated the linear mixed-effects model, but now studying the main effect (marked bold in Equation 2). The main effect reveals whether prenatal infection, irrespective of time, is related to brain morphology. Second, generally, studies have suggested to be cautious in adjusting for ICV in repeated-measures models (Mills et al., 2016; Herting et al., 2018). Hence, we did not adjust for it in our primary analyses. However, as there is no consensus, as a second sensitivity analysis, we repeated the linear mixed-effects model of our primary research question between prenatal infection and child brain morphology over time additionally adjusting for ICV. Third, because results in our trimester-specific analysis could be confounded by an infection in the other trimester, and thus to better understand whether a specific trimester is a sensitive period, we performed a third sensitivity analysis. For an FDR-significant finding in the trimester-specific analyses (after correction for multiple testing), we repeated the analysis but also additionally adjusting for the interaction between time and the infection sum scores from the other trimesters. Fourth, to compare the effect sizes of our significant findings to another known risk factor that may influence child brain development, we have run a sensitivity analysis between maternal education (as proxy for socioeconomic status) and the FDR-significant brain regions. In this model we investigated the interaction between maternal education and time. Fifth, for the FDR-significant findings, we tested potential moderation by environmental factors that may exacerbate or dampen associations between prenatal infection exposure and brain morphology, including prenatally assessed maternal education, maternal psychopathology, maternal alcohol use, and postnatal life events (child), postnatal direct victimization (child). Sixth, to assess whether FDR-significant findings were not driven by the maternal inflammatory status, we reran the analyses additionally adjusting for maternal immune conditions such as diabetes, preeclampsia and pregnancy induced hypertension. Seventh, we further applied two sensitivity analyses to determine the effect of severity of infections on our FDR-significant findings. In the first analysis we repeated the linear mixed-effects models with the infection sum score but now using nine domains instead of ten, as we omitted fever. We also tested the interaction between the infection sum score (without the fever domain) and fever in the association between infections during pregnancy and the FDR-significant brain findings. Eighth, as an additional sensitivity analysis we tested the effect of child age as time variable instead of neuroimaging visit as time variable for the FDR-significant findings. Ninth, we tested the effect of modeling the interaction between prenatal infections and time as a non-linear term by using natural cubic splines for the FDR-significant brain findings. Finally, tenth, we explored the role of child sex as moderator in our main analyses, given that there is evidence that suggests that the placental immune markers may differentially respond to immune activation in the mother, specifically suggesting that the male placental unit may be more susceptible (Hunter et al., 2019).

$$\begin{array}{l} {\text{Y}}_{\text{ij}}\;\text{\textasciitilde{}}\;\text{prenatal}\;{\text{infection}}_{i}\;\text{+}\;{\text{time}}_{\text{ij}}\;\text{+}\;\text{child}\;{\text{sex}}_{\text{i}}\;\text{+}\;\text{maternal}\;{\text{age}}_{\text{i}}\;\text{+}\\ \text{maternal}\;\text{national}\;{\text{background}}_{\text{i}}\;\text{+}\;\text{household}\;{\text{income}}_{\text{i}}\;\text{+}\;\text{maternal}\;{\text{education}}_{\text{i}}\\ \text{+}\;\text{maternal}\;\text{tobacco}\;{\text{use}}_{\text{i}}\;\text{+}\;\text{maternal}\;\text{prenatal}\;{\text{psychopathology}}_{\text{i}}\\ \text{+}\;{\text{μ0}}_{\text{i}}\;\text{+}\;{\text{ε}}_{\text{ij}} \end{array}$$

Equation 2. Linear mixed-effects model. The i subscript indicates the subject, the j subscript indicates the timepoint. The term ‘Yij’ indicates the individual child brain morphology outcomes. The ‘prenatal infection_i_’ is the term of interest of which the coefficient shows the average effect of prenatal infection on child brain morphology over time (i.e., assuming the association is not different over time). Time is defined as time of MRI scan. The term μ0_i_ indicates the random intercept. Prenatal infection is entered as a continuous variable in this model. In this model we model both the main effect as well as the interaction effect; but only the interaction effect is interpreted.

To account for confounding bias, we corrected all models for child sex, maternal age, maternal national background, household income, maternal education, maternal tobacco use, and maternal prenatal psychopathology (see Confounders and Covariates section above). Given the nature of long-term prospective studies, loss to follow-up occurs as retention of all participants is not always possible. This results in potentially non-random selection of observations and can lead to selection bias in regression coefficients (both over and underestimates in associations) (Dijkzeul et al., 2024). In order to mitigate the effects of this bias, we conducted inverse-probability weighting (Weuve et al., 2012). This method enables us to give weights to each individual depending on their probability of being selected in the sample, e.g., individuals with a lower probability to be selected in the study sample will get a higher weight to act, in a way, as stand ins for similar individuals who were lost to follow-up. First, we conducted a non-response analysis to obtain differences in our subsample compared to the whole Generation R cohort at baseline. Then, the probability of remaining in the study (not being lost to follow-up) was calculated with the covariate balancing propensity score method (CBPS package), after which weights were calculated by taking the inverse of those probabilities. The following predictor variables were used to calculate weights: maternal age, maternal national background, maternal smoking, maternal psychopathology, prenatal infection total score, sex child, maternal education, household income, and SSRI. We further used the CBCL total behavioral problems score and the total score of the SRS when the child was six years old. The reason for specifically including SSRI, CBCL and SRS as predictors was because ~ 50 % of the imaging sample at T1 was oversampled from the underlying cohort based on prenatal SSRI usage and a higher score on both behavior traits. The other 50 % of the sample at T1 was randomly selected from the underlying cohort. Hence, by including these additional variables as predictors for the IPW, we wanted to ensure that the distribution of participants with these phenotypes was the same.

Moreover, to account for missing data in covariates (max 6 % missing on a given covariate), we applied multiple imputation using chained equations (*mice* package) to impute 30 datasets and 50 iterations (Buuren and Groothuis-Oudshoorn, 2011). Missingness in outcomes was accounted for with restricted maximum likelihood in the linear mixed-effects models. Given the different use of scanners and the application of multiple imputation, we repeated our analyses for the FDR-significant findings with data from neuroimaging waves 2 and 3 (same scanner) only and we repeated analyses for our FDR-significant findings with complete cases only as a robustness check. And finally, to account for the multiple tests, we applied the false discovery rate – Benjamini Hochberg (FDR-BH) correction within each research aim. Of note, for the secondary analysis investigating potential trimester-specific associations in three separate models, we adjusted for each trimester model individually. An FDR-corrected p-value below 0.05 was used as significance threshold. Of note, a power analysis can be found in Supplemental Text B. All statistical analyses were performed with R Statistical Software (version 4.1.2; R Development Core Team); the script used for this project can be found on: https://github.com/ajsuleri/Prenatal_infection_child_brain_development.

## 3 Results

### 3.1 Descriptive results

**Fig. 2:**
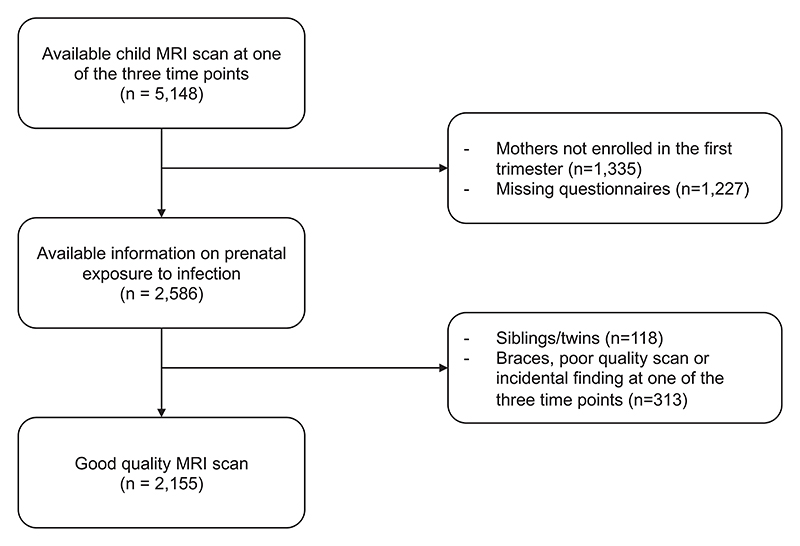
Flowchart depicting the selection of study participants.

After applying our inclusion and exclusion criteria, data from 2,155 mother–child dyads were available for analyses (Fig. 2). The average age of the mother at enrollment was 31 years and 56.1 % of the mothers had higher education. The median prenatal infection sum score was 3 for the whole pregnancy (which corresponds to three reported infections) (Fig. 1). More demographic information about the participants can be found in Table 1. The results of the nonresponse analysis can be found in Supplemental Text C.

### 3.2 Total infections across pregnancy and child brain development

Our primary analyses showed that prenatal exposure to a higher number of infections during the whole pregnancy was associated with a volume increase (as opposed to a decrease in those exposed to fewer infections) in temporal pole volume over time (β = 0.060, 95 % CI 0.010 – 0.110, p_uncorrected_ = 0.020), a faster increase in the cerebellum volume over time (β = 0.038, 95 % CI 0.003 – 0.073, p_uncorrected_ = 0.034), and a slower increase in the thalamus volume over time (β = -0.035, 95 % CI –0.062 – –0.008, p_uncorrected_ = 0.011) (Table 2). Importantly, these associations did not survive multiple testing correction.

### 3.3 Trimester-specific effects of infections on child brain development

**Fig. 3:**
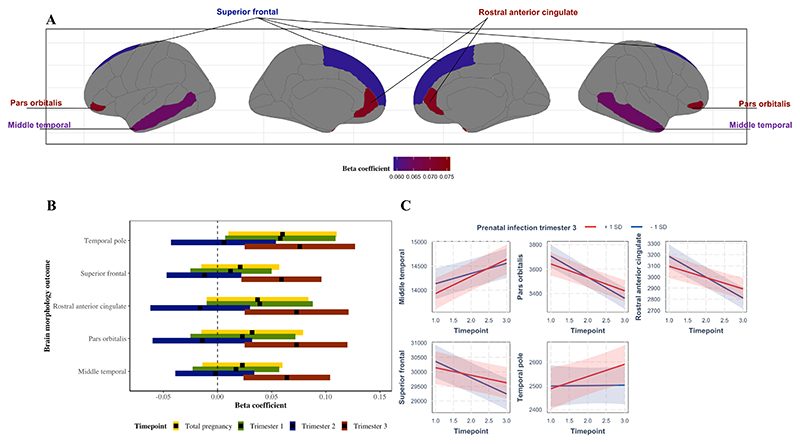
Prenatal infection and changes in child brain morphology; significant (p_fdr_ < 0.05) results. Plot 3A depicts the neuroanatomical position of the significant regions in trimester 3. Plot 3B shows a forest plot for the significant brain regions in trimester 3, but also the effect estimates and confidence intervals of the same regions in trimesters 1 and 2. In plot 3C, for visualization purpose only, prenatal exposure to infection is dichotomized into ‘+1 SD’ and ‘-1 SD’ groups, and timepoints 1, 2 and 3, indicate child’s mean age 8, 10 and 14, respectively.

**Fig. 4:**
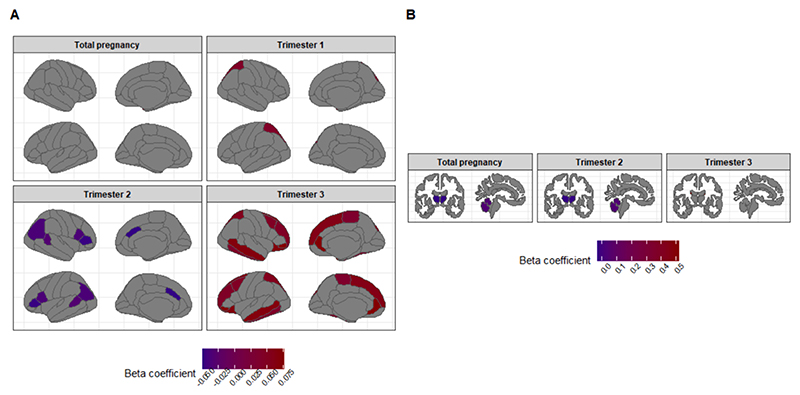
Brain anatomy plot with significant (p_uncorrected_ < 0.05) cortical and subcortical brain regions.

Our secondary analyses investigating trimester-specific effects showed no significant association between infections in the first trimester or second trimester and changes in brain morphology after multiple testing correction (Tables 3-4). Infections in the third trimester were associated with a faster increase in volume over time in the middle temporal gyrus (β = 0.064, 95 % CI 0.024 – 0.104, p = 0.002) (Fig. 3, Figure S4, Table 5). Moreover, we observed a slower decrease in volume over time in the pars orbitalis (β = 0.073, 95 % CI 0.025 – 0.120, p = 0.003), rostral anterior cingulate (β = 0.073, 95 % CI 0.025 – 0.121, p = 0.003), superior frontal gyrus (β = 0.059, 95 % CI 0.022 – 0.096, p = 0.002) (Fig. 3, Figure S4, Table 5). We further observed an increase of volume over time in temporal pole (β = 0.076, 95 % CI 0.025 – 0.127p = 0.003 (Fig. 3, Figure S4, Table 5). The nominal significant results before multiple testing correction can be seen in Fig. 4 as well as Figures S5-8 and highlight several regions in mainly the frontal and temporal lobe, cerebellum, and thalamus. A visualization of all results can be found in Figure S9.

### 3.4 Sensitivity analyses

The first sensitivity analysis, in which we studied the main effect of prenatal infection (i.e., not including the interaction with time), showed no significant associations after multiple testing correction (Table S1). Next, we evaluated the impact of ICV on primary analyses. After additionally adjusting for ICV in the longitudinal association between prenatal infection (total pregnancy) and brain morphology over time, there were no significant associations after multiple testing correction (Table S2). Aside from this, results remained consistent with primary analyses (Table S2). Moreover, to explore the specificity of the trimester-specific results, models were mutually adjusted for each trimester infection score. After mutually adjusting for the other infection sum scores, the association between infections during pregnancy in the third trimester were no longer associated with changes in temporal pole volume over time (Table S3).

Complete case analysis yielded similar effect sizes (Table S4). We observed no evidence for an association between maternal education and changes in brain development in the five FDR-significant brain regions (Table S5). There was no statistical moderation of maternal education, maternal psychopathology, maternal alcohol use, and postnatal life events, or postnatal direct victimization in the association between prenatal infections in trimester 3 and the five FDR-significant brain regions (Table S6). Moreover, the FDR-significant results remained after additionally adjusting for maternal immune conditions (Table S7). When we repeated our analyses for the significant findings with the infection sum score without fever, we observed that all regions except for pars orbitalis remained significant (Table S8). Furthermore, there was no significant interaction between the infection sum score without fever and fever (Table S9). Results remained similar after modeling the mixed-effects models with child age instead of neuroimaging visit as time variable, except for the temporal pole which was no longer significant (Table S10). When adding splines to the model, the model fit only changed marginally (i.e., 0.1–1 % change in AIC/BIC) after adding splines (Table S11). Further, most of the spline terms were non-significant (Table S12), suggesting that the linear fits were preferable. Child sex was not a moderator in the main analyses, as the interaction effect for sex was non-significant (Table S13). Given that the scanner between neuroimaging visit 1 was different than visits 2 and 3 and we cannot adjust for the scanner, we also show the results for the FDR-significant regions using data from only visit 2 and 3 (Table S14). Finally, to better understand our significant results, we conducted post-hoc linear regression analyses for each time point individually focusing on the significant brain regions, aiming to facilitate the interpretation of our findings (Table S15).

## 4 Discussion

In this prospective population-based study, we examined the longitudinal association between prenatal exposure to infections and child brain development from early childhood to adolescence (mean age 8 to 14 years) using repeated measures of neuroimaging. After multiple testing correction, we observed that prenatal exposure to infections in the third trimester was associated with time-varying changes in brain morphology, particularly in frontotemporal regions of the child’s brain. We did not find a stable (i.e., time-invariant) association between prenatal exposure to infections and child brain morphology over time. There was no moderation effect of various environmental factors (related to maternal lifestyle and psychopathology) on the association between prenatal infections and changes in brain morphology over time. There was also no significant interaction between infections and fever for the significant brain regions; however, sensitivity analyses suggest that fever may explain some, but not all, of the associations. Lastly, we found no evidence that child sex was a moderator in the association between prenatal infections and changes in brain morphology over time.

### 4.1 Interpretation of brain regions and timing of effect

After correction for multiple testing, we found that prenatal exposure to infections during the third trimester was associated with repeated measures of several structures of the child’s brain: middle temporal gyrus (faster increase over time), pars orbitalis (slower decrease over time), rostral anterior cingulate (slower decrease over time), superior frontal gyrus (slower decrease over time), and temporal pole (increase over time). These frontotemporal regions have been implicated in social, emotional, and cognitive processes (Hoffmann, 2013; Jin et al., 2022; Wong and Gallate, 2012). This is of interest given that prior studies in the field have suggested associations between prenatal exposure to infections and the development of emotional, social, and cognitive problems in children (Han et al., 2021; al-Haddad et al., 2019; Khandaker et al., 2013; Suleri et al., 2023; Kwok et al., 2022). While our sensitivity analyses revealed persistent associations with these frontotemporal regions even after mutual adjustment for each trimester, it is important to exercise caution when interpreting trimester-specific effects. The overlapping confidence intervals observed across all trimester results, particularly notable in trimesters one and three, imply a potential lack of differential effects between these periods, despite the statistically significant results observed in the third trimester.

There may also be neurobiological considerations. Notably, the third trimester marks an important period in which immune cells colonize and multiple neurodevelopmental processes take place (Estes and McAllister, 2016). Moreover, there is a notable expansion in the brain’s dimensions, accompanied by the ongoing refinement of neural connections (Andescavage et al., 2017). These transformations are believed to play a pivotal role in shaping advanced cognitive abilities throughout childhood and into adulthood (Bouyssi-Kobar et al., 2016). Hence, this period may render the developing brain more susceptible to external influences, including prenatal infections. In addition, the frontal and temporal lobes specifically undergo substantial growth and maturation in the third trimester. The timing of our observed morphometric changes may reflect the increased vulnerability of these regions during this specific developmental window. Another explanation of our timing effects may be due to the intricate interplay between the maternal and fetal immune system during the third trimester. Hence, immune responses and inflammation during this period may have more pronounced effects on neural development. However, we acknowledge the current gaps in understanding prenatal infections and their potential trimester-specific impact on neurodevelopment. Especially because earlier human studies have mostly identified the first trimester as potential sensitive window for the effects of prenatal infections (Guma et al., 2019). Interestingly, two rodent studies have explored the impact of the bacterial mimetic LPS and the influenza virus H2N3 on the developmental outcomes of neonatal offspring in the rhesus monkey (Guma et al., 2019). The results revealed that offspring exposed to LPS at 17 weeks gestation exhibited heightened global white matter volume and increased gray matter thickness in the right parietal and frontal lobes, while concurrently showing decreased gray matter volume in the medial temporal lobe (Guma et al., 2019). This may suggest that even in the presence of an infection in the second trimester, this may have a modest impact on child brain development and that insults during early or late gestation may be more important for fetal brain development. However, given the observational nature of the data, this is purely a speculative discussion on the potential interpretation of the findings and further investigations are needed to robustly identify potential sensitive windows for the effects of prenatal infections during pregnancy on the offspring’s brain in humans. Of note, after mutually adjusting for infections in the first and second trimesters, the association between infections during pregnancy and the temporal pole was no longer significant, and associations across trimesters had overlapping confidence intervals, limiting our ability to interpret associations as specific to a particular trimester.

### 4.2 Comparison of our findings with prior animal studies

Our study adds to the extensive body of animal literature that suggests a potential association between prenatal infection and child brain development (Guma et al., 2019). Although there are clear differences in developmental timing and anatomy between rodents and humans, we observed interesting parallels to pre-existing animal research across diverse gestational windows. In contrast to our findings, rodent studies have observed that infections in early gestation were typically associated with accelerated brain growth, and increased frontal and occipital lobe volume (driven by white matter volume expansion) in the first two years postnatally (Guma et al., 2019). Infections in early gestation were furthermore associated with larger ventricle volume, and lower fractional anisotropy in regions such as the amygdala and anterior cingulate (Guma et al., 2019). Rodent studies using a similar developmental period that investigated infections in mid gestation, observed no associations with volume changes in total brain volume, lateral ventricles, or the hippocampus in adolescent rats, but did observe associations with prefrontal cortex volume, anterior cingulate volume, or the hippocampus in early and late adulthood mice (Guma et al., 2019). Furthermore, rodent studies that focus on infections in late gestation have typically found widespread changes in white matter microstructure in *peri*-adolescent and adult rats, as well as changes in regions such as the forebrain, thalamus, hypothalamus, cerebellum, and hippocampus (Guma et al., 2019). Of note, the third trimester in humans includes processes such as synaptogenesis, gliogenesis and myelination, all of which begin postnatally in mice studies (Guma et al., 2019). However, non-human primates do align more closely in neurodevelopment to humans with respect to comparability and developmental timing. As such, non-human primate studies investigating infections during late gestation typically found changes in frontal and temporal lobes in both gray and white matter in neonatal offspring (Guma et al., 2019). Hence, although we do not observe any associations in early gestation in contrast to prior animal studies, intriguingly, our findings in late gestation in the frontal and temporal lobes align with earlier established research in animal studies, given that animal studies have consistently implicated these regions as vulnerable targets across different developmental periods (Guma et al., 2019; Guma et al., 2021). However, given the overlapping confidence intervals as mentioned in the prior paragraph, it is difficult to make concrete conclusions about the potential effects of the timing of infection.

Additionally, animal studies have delved into various cellular mechanisms that may underlie the association between prenatal infections and child brain development. A systematic review suggests that epigenetic and transcriptomic alterations in specific brain regions might link prenatal infections to child neurodevelopment, echoing potential associations observed in our study (Woods et al., 2021). Furthermore, microglial activation (i.e., differentially programmed towards a pleiotropic phenotype) following prenatal immune activation might lead to excessive neurogenesis, a phenomenon observed in autism spectrum disorders (Loayza et al., 2023). Another recent human brain organoid study adds further clarity – as they identified selective vulnerability of radial glia cells after prenatal maternal immune activation, suggesting a potential shift towards a younger developmental state. This may align with our findings that implies potential disruptions in typical brain development trajectories following prenatal infections (Sarieva et al., 2023).

### 4.3 Interpretation of longitudinal patterns

To better understand the results, it is important to put them in context of typical neurodevelopment as cortical maturation follows an inverted U shape curve (Di Martino et al., 2014; Sudre et al., 2018). Different brain regions (regardless of prenatal exposure to infections) develop at different rates and may show different typical direction of effects depending on their developmental period (i.e., faster versus slower growth over time). As we examined children within the average age range of 8 to 14 years, a pivotal period marked by ongoing maturation of frontotemporal regions extending into early adulthood, our significant findings were in specific areas in the frontal and temporal lobes (Mills and Tamnes, 2014). This result may be explained by the prolonged development of these regions compared to other e.g., sensorimotor areas, which tend to mature at an earlier age.

Despite limited data from repeated-measure neuroimaging studies in humans, animal models with serial measurements exhibit a parallel longitudinal pattern, suggesting catch-up growth in mice offspring exposed to (severe) prenatal infections (Crum et al., 2017; Guma et al., 2021). Hence, one interpretation of our findings may be that children who were exposed to infections prenatally are also showing ‘catch-up growth’; a delay in typical brain growth compared to the children who were not exposed to infections prenatally which then normalizes over time (Di Martino et al., 2014; Desmond and Casale, 2017). This implies that these children exhibit accelerated growth (in line with the typical trajectory) that eventually allows the trajectory to converge with the expected brain volume of unexposed children. This accelerated growth might reflect compensatory mechanisms attempting to mitigate the effects of prenatal infections on brain development. For example, catch-up growth could manifest as accelerated neuronal maturation, synapse formation, and myelination, enabling the brain to compensate for any initial setbacks and bring the brain development back on track or closer to typical developmental trajectories. However, the data from the present study may not be able to entirely support such an interpretation of catch-up growth. First, the age-range is relatively narrow. Future research encompassing a wider age range from early childhood into early adulthood (e.g., until the age of 25 years), may then find that these frontotemporal regions have largely normalized by this age and would be able to confirm whether we observe catch-up growth. Second, single time point analyses in the present study show differences in the direction of effect sizes in the analysis at the first neuroimaging visit (i.e., negative direction) compared to the second and third neuroimaging visit (i.e., both showing positive directions). However, these effects, perhaps due to use of single time point data and resulting smaller sample sizes, were non-significant.

Given this, as catch-up growth suggests that there is a larger deviation at baseline that would need to be caught-up from, alternative explanations should also be considered. Catch-up growth is furthermore typically associated with improvements in cognitive, emotional, and behavioral functioning, and our prior work may argue against catch-up growth. Prior work from our group – in line with other literature–namely demonstrated that prenatal exposure to infections in the same cohort was associated with more internalizing and externalizing symptoms in adolescence (al-Haddad et al., 2019; Suleri et al., 2023; Suleri et al., 2023). Considering the role that the frontal and temporal lobe may play in the neurobiology of internalizing and externalizing behavioral problems (Desikan et al., 2006), it may also be that there are longitudinal deviations in these areas after prenatal exposure to infections. This would be in line with the previously mentioned human brain organoid study that observed a longitudinal shift in the developmental curve (Sarieva et al., 2023). Hence, that would suggest that the observed changes do not indicate a temporary ‘lag’ in brain development which results in a complete return to typical brain development, but instead indicates a long-term adjustment in growth pattern. Of note, even if these deviations were to be caught up with later, it could be that the catch-up growth in the brain is not fully sufficient to completely alleviate the underlying neurobiological or genetic factors (specifically gene x environment interactions) contributing to the psychopathology. Alternatively, another plausible explanation may be that these changes represent nuanced/subtle shifts in the overall growth pattern. To clarify, instead of catch-up growth where the development initially falls behind and then normalizes later, this alternative interpretation suggests a consistent, though smaller, deviation from the expected development. This deviation may not necessarily mean a complete departure from normalcy, but rather indicate a distinct trajectory ‘locally’ in the developmental curve (i.e., within a restricted age-range) within the normal variations of neurodevelopment.

Furthermore, an important methodological constraint that should also be considered, is the use of a different scanner type between neuroimaging assessment 1 versus neuroimaging assessments 2 and 3. When we repeated our analyses in only the last two neuroimaging assessments, our effect sizes attenuated but remained mostly in the same direction; however, the results were no longer significant. While this may also be due to the shorter developmental window, only including the (pre) adolescent period and not the childhood period, as well as the reduction in sample size, it cannot be ruled out that scanner type could contribute to the results. Of note, given that we are examining relative change over time (as a function of exposure status) as opposed to absolute change over time, the potential for the change in MRI scanning fully accounting for the associations with exposure to infection is unlikely.

And finally, the significance of the findings is tempered given that the effect sizes observed are notably small. Hence, the interpretation of the results of our study warrants caution. This has also been outlined in prior literature that studies with increased sample size observe smaller effect sizes of which it becomes questionable whether these associations are meaningful (Smith and Nichols, 2018). It is important to consider these effect sizes in the broader context of multifactorial prenatal influences on child neurodevelopment. Small effect sizes may be relevant for the etiology but are unlikely to be relevant for prediction or biomarker work where factors are needed that explain a large part of the variance (Marek et al., 2022).

### 4.4 Non-FDR findings

We further observed some findings in other regions of interest that contained important values in their confidence intervals, despite those regions not reaching statistical significance after multiple testing correction. These regions were in the frontotemporal part of the brain (e. g., caudate anterior cingulate, fusiform, or rostral middle frontal), in the subcortex (such as the thalamus and caudate), and in the cerebellum, that were modestly related to prenatal exposure to infections. Given the confidence intervals observed, the fact that these brain structures have also been observed in prior animal studies (Guma et al., 2019), and the ongoing debates on multiple testing correction and the use of p-values (i. e., striking a balance between avoiding type I errors to minimize false positive findings but maintaining sensitivity to true associations; so also minimizing false negative findings) (Marek et al., 2022; Feise, 2002), these regions may still be potential targets to investigate in future studies.

### 4.5 Discrepancies with prior studies

Unlike prior studies that identified the hippocampus and amygdala as potential targets in the toddler’s brain after prenatal exposure to inflammation (Graham et al., 2018; Rudolph et al., 2018; Rasmussen et al., 2022), we did not observe these brain regions. One potential reason is that these brain regions were already largely normalized at the mean age of 10 and are thus only observed in the toddler’s brain (Uematsu et al., 2012). Alternatively, as the prior studies investigated inflammation instead of infections, this could be a potential reason underlying the discrepancies between our studies. Although (severe) infections may lead to inflammation, there are also other causes of inflammation such as obesity or autoimmune diseases (Han et al., 2021).

### 4.6 Strengths and limitations

Our study has several strengths and limitations. The use of a large socio-demographically and culturally diverse, prospective population-based cohort (N ≈ 2,200), in which we investigated a broad range of infections during each trimester of pregnancy and used serial measurements of neuroimaging outcomes is a clear strength. We further made corrections for important confounding factors, attrition bias, and multiple testing. However, information on prenatal infections was collected once per trimester and no blood was drawn at time of infection to verify the prenatal infections quantitatively/objectively. The self-reported questionnaires are subject to recall and reporter bias; yet the infections were shortly recalled after each trimester limiting recall bias. Moreover, another advantage of self-report questionnaires is that rather than a visit to the research center for biological measurements that may have a short half-life, these questionnaires could be filled in at home during any moment, and thus may be less likely to suffer from healthy volunteer (selection) bias, whereby participants are less likely to attend a research visit when experiencing an infection. Non-response analyses in this study revealed that there were some differences between the mothers included in the analyses and all the mothers from the original baseline cohort. To address the potential selective loss to follow-up, we imputed covariates, and we applied inverse-probability weighting to address potential selection bias. Additionally, as our study contains observational data, the degree to which we can make causal inferences is limited. Moreover, another limitation of our study is that we were unable to assess the severity of the infections, other than the effect of fever.

Future studies should further investigate the effect of severity and chronicity of infection on the association between prenatal exposure to infections and child brain development. Furthermore, a limitation is that a different scanner was used at the first imaging wave (child mean age 8 years) as compared to the other two imaging waves (child mean ages 10 and 14 years), and we were unable to account for that in our statistics. Another limitation of the study is that we limited analyses to the Desikan-Killiany parcellation, which is an anatomical parcellation of the brain. It is possible that a functional parcellation would offer new and important information. Finally, we followed a hierarchical approach, where we ran main analyses, and only performed sensitivity analyses on FDR-significant findings to ensure robustness. However, this approach could increase type II errors, where some associations could be missed.

### 4.7 Conclusion

In conclusion, in this large population-based study, we observed that prenatal exposure to infections in the third trimester were associated with various frontotemporal regions in the child’s brain over a mean time span of 8 to 14 years. Although we only observed associations with infection exposure in the third trimester, the evidence for a trimester-specific effect is limited and it is likely that the effect of prenatal infections during pregnancy is general. We further did not observe time-invariant associations between prenatal infections and child brain morphology across childhood. Taken together, our study adds onto the growing evidence base in especially animal studies – but also a few single time-point human studies – that prenatal exposure to infections may affect child brain development in especially regions of the frontal and temporal lobe.

## Supplementary Material

Supplementary materials

## Figures and Tables

**Table 1:** Demographic information participants.

	Sample (n = 2,155)
**Maternal characteristics**	
Age mother at enrollment (mean, SD)	31.0 (4.5)
*Infection sum score (mean, SD)*	
Total prenatal infection score	3.0 (2.2)
Trimester 1 infection score	1.1 (1.0)
Trimester 2 infection score	1.0 (1.0)
Trimester 3 infection score	1.0 (1.0)
*National background (n, %)*	
Dutch	1,412 (65.5)
Non-Dutch	733 (34.0)
Missing	10 (0.5)
*Maternal education (n, %)*	
Low	96 (4.5)
Intermediate	831 (38.6)
High	1,210 (56.1)
Missing	18 (0.8)
*Household income (n, %)*	
< €2200 per month	680 (31.6)
> €2200 per month	1,386 (64.3)
Missing	89 (4.1)
*Smoking habits (n, %)*	
Never smoked during pregnancy	1,649 (76.5)
Smoked until pregnancy was known	187 (8.7)
Continued smoking in pregnancy	295 (13.7)
Missing	24 (1.1)
*Prenatal SSRI usage (n, %)*	
During pregnancy at any trimester	10 (0.5)
Early pregnancy only	11 (0.5)
Only before pregnancy	11 (0.5)
No use	2059 (95.5)
Missing	64 (3.0)
**Child characteristics**	
Child’s sex, female (%)	1,103 (51.2)
CBCL total behavioral problems sum score (at age 6) (mean, SD)	18.8 (15.9)
SRS weighted total sum score (at age 6) (mean, SD)	0.21 (0.23)

**Table 2:** Prenatal infection and child brain volumes (interaction effect).

	Standardized β-coefficient	95 % Confidence interval		B-coefficient	P-value
Total brain volume	0.026	−0.001	0.052	1371.542	0.062
Banks of the superior temporal sulcus	−0.024	−0.053	0.004	−4.951	0.089
Caudal anterior cingulate	−0.003	−0.037	0.031	−0.541	0.875
Caudal middle frontal	0.023	−0.014	0.060	14.231	0.220
Cuneus	−0.008	−0.033	0.016	−2.460	0.497
Entorhinal	0.002	−0.037	0.041	0.417	0.915
Fusiform gyrus	0.017	−0.016	0.050	10.930	0.316
Inferior parietal	−0.004	−0.036	0.028	−4.639	0.797
Inferior temporal	0.023	−0.013	0.059	20.516	0.204
Isthmus cingulate	−0.009	−0.029	0.011	−1.961	0.391
Lateral occipital	0.008	−0.016	0.032	7.391	0.521
Lateral orbitofrontal	0.034	−0.014	0.082	22.963	0.167
Lingual	−0.013	−0.035	0.010	−6.577	0.266
Medial orbitofrontal	0.005	−0.040	0.050	2.249	0.841
Middle temporal	0.032	−0.007	0.070	27.956	0.108
Parahippocampal	−0.006	−0.040	0.028	−0.902	0.714
Paracentral	0.003	−0.028	0.035	0.864	0.831
Pars opercularis	−0.007	−0.035	0.021	−2.534	0.631
Pars orbitalis	0.032	−0.015	0.079	8.442	0.186
Pars triangularis	−0.029	−0.062	0.004	−10.636	0.085
Pericalcarine	−0.008	−0.042	0.025	−1.747	0.637
Postcentral	0.012	−0.014	0.039	8.199	0.370
Posterior cingulate	0.003	−0.022	0.027	0.738	0.814
Precentral	0.000	−0.035	0.034	−0.320	0.982
Precuneus	0.007	−0.022	0.036	5.426	0.640
Rostral anterior cingulate	0.037	−0.010	0.084	11.291	0.124
Rostral middle frontal	0.011	−0.029	0.052	16.945	0.579
Superior frontal	0.021	−0.015	0.057	33.280	0.263
Superior parietal	0.023	−0.014	0.061	24.242	0.228
Superior temporal	0.003	−0.032	0.039	2.778	0.846
Supramarginal	0.004	−0.025	0.034	3.966	0.782
Frontal pole	0.024	−0.021	0.069	3.069	0.296
Temporal pole	0.060	0.010	0.110	14.431	0.020*
Transverse temporal	−0.013	−0.038	0.012	−1.129	0.315
Insula	0.024	−0.007	0.054	10.488	0.124
Cerebellum	0.038	0.003	0.073	113.957	0.034*
Amygdala	0.016	−0.024	0.056	1.727	0.421
Hippocampus	−0.001	−0.028	0.025	−0.243	0.919
Caudate	0.027	−0.013	0.066	7.357	0.188
Putamen	0.013	−0.021	0.047	3.825	0.459
Thalamus	−0.035	−0.062	−0.008	−11.635	0.011*
Pallidum	0.025	−0.013	0.064	2.724	0.195

* p < 0.05.

** FDR-BH corrected p < 0.05.

**Table 3:** Prenatal infection trimester 1 and child brain volumes (interaction effect).

	Standardized β-coefficient	95 % Confidence interval		B-coefficient	P-value
Total brain volume	0.026	−0.002	0.053	2893.389	0.068
Banks of the superior temporal sulcus	−0.014	−0.044	0.015	−6.175	0.331
Caudal anterior cingulate	0.025	−0.010	0.060	10.508	0.158
Caudal middle frontal	0.031	−0.007	0.069	40.197	0.110
Cuneus	−0.010	−0.035	0.015	−6.184	0.433
Entorhinal	0.014	−0.026	0.054	5.913	0.486
Fusiform gyrus	0.018	−0.016	0.053	25.051	0.296
Inferior parietal	0.014	−0.019	0.046	31.909	0.418
Inferior temporal	0.013	−0.024	0.050	25.104	0.477
Isthmus cingulate	−0.007	−0.028	0.014	−3.055	0.540
Lateral occipital	0.018	−0.007	0.043	34.899	0.165
Lateral orbitofrontal	0.035	−0.015	0.084	49.736	0.172
Lingual	0.003	−0.020	0.026	2.886	0.823
Medial orbitofrontal	0.026	−0.020	0.073	27.229	0.269
Middle temporal	0.017	−0.023	0.057	31.674	0.405
Parahippocampal	−0.007	−0.042	0.028	−2.027	0.705
Paracentral	−0.005	−0.038	0.028	−2.655	0.764
Pars opercularis	0.008	−0.022	0.037	5.843	0.611
Pars orbitalis	0.023	−0.025	0.072	13.067	0.347
Pars triangularis	−0.023	−0.057	0.011	−17.69	0.188
Pericalcarine	−0.011	−0.046	0.023	−5.209	0.517
Postcentral	0.012	−0.016	0.039	16.765	0.402
Posterior cingulate	0.014	−0.012	0.039	7.181	0.293
Precentral	0.018	−0.018	0.054	31.253	0.322
Precuneus	0.018	−0.012	0.048	30.049	0.237
Rostral anterior cingulate	0.039	−0.010	0.088	25.119	0.116
Rostral middle frontal	0.018	−0.024	0.061	57.470	0.393
Superior frontal	0.012	−0.025	0.050	42.033	0.519
Superior parietal	0.041	0.002	0.079	89.817	0.040*
Superior temporal	0.008	−0.029	0.044	12.824	0.680
Supramarginal	0.020	−0.010	0.051	41.227	0.189
Frontal pole	0.013	−0.033	0.059	3.469	0.585
Temporal pole	0.058	0.007	0.109	29.516	0.027*
Transverse temporal	−0.010	−0.035	0.016	−1.802	0.462
Insula	0.019	−0.013	0.050	17.419	0.244
Cerebellum	0.023	−0.013	0.060	148.909	0.211
Amygdala	0.018	−0.024	0.059	3.924	0.405
Hippocampus	−0.008	−0.036	0.020	−2.952	0.574
Caudate	0.026	−0.015	0.067	15.197	0.211
Putamen	0.018	−0.018	0.054	11.269	0.319
Thalamus	−0.013	−0.041	0.015	−8.944	0.373
Pallidum	0.019	−0.021	0.058	4.281	0.350

* p < 0.05.

** FDR-BH corrected p < 0.05.

**Table 4:** Prenatal infection trimester 2 and child brain volumes (interaction effect).

	Standardized β-coefficient	95 % Confidence interval		B-coefficient	P-value
Total brain volume	0.001	−0.025	0.027	143.957	0.927
Banks of the superior temporal sulcus	−0.032	−0.058	−0.005	−14.245	0.020*
Caudal anterior cingulate	−0.050	−0.082	−0.018	−21.785	0.002*
Caudal middle frontal	−0.018	−0.053	0.017	−24.504	0.313
Cuneus	−0.018	−0.040	0.005	−11.498	0.129
Entorhinal	−0.012	−0.049	0.024	−5.479	0.505
Fusiform gyrus	−0.012	−0.043	0.020	−16.772	0.467
Inferior parietal	−0.038	−0.069	−0.008	−95.183	0.014*
Inferior temporal	0.006	−0.028	0.039	10.906	0.748
Isthmus cingulate	−0.013	−0.032	0.006	−6.465	0.173
Lateral occipital	−0.009	−0.032	0.014	−18.940	0.433
Lateral orbitofrontal	−0.010	−0.056	0.037	−14.693	0.684
Lingual	−0.013	−0.034	0.007	−15.551	0.206
Medial orbitofrontal	−0.034	−0.078	0.010	−36.906	0.130
Middle temporal	−0.002	−0.039	0.034	−4.692	0.898
Parahippocampal	−0.030	−0.062	0.002	−9.390	0.067
Paracentral	−0.016	−0.045	0.014	−8.757	0.300
Pars opercularis	−0.037	−0.064	−0.011	−30.173	0.006*
Pars orbitalis	−0.014	−0.060	0.032	−8.203	0.554
Pars triangularis	−0.048	−0.079	−0.016	−38.633	0.003*
Pericalcarine	−0.017	−0.048	0.015	−8.038	0.298
Postcentral	−0.005	−0.030	0.020	−8.010	0.677
Posterior cingulate	−0.018	−0.041	0.005	−9.845	0.133
Precentral	−0.028	−0.061	0.005	−50.433	0.099
Precuneus	−0.022	−0.050	0.005	−39.344	0.107
Rostral anterior cingulate	−0.016	−0.062	0.030	−10.981	0.487
Rostral middle frontal	−0.022	−0.061	0.017	−72.372	0.269
Superior frontal	−0.012	−0.047	0.022	−43.780	0.487
Superior parietal	−0.030	−0.067	0.006	−70.819	0.101
Superior temporal	−0.023	−0.057	0.010	−41.317	0.171
Supramarginal	−0.021	−0.049	0.007	−44.132	0.149
Frontal pole	0.005	−0.038	0.048	1.356	0.829
Temporal pole	0.006	−0.043	0.054	3.160	0.811
Transverse temporal	−0.019	−0.042	0.005	−3.622	0.125
Insula	0.012	−0.017	0.040	11.329	0.428
Cerebellum	0.037	0.004	0.071	249.103	0.029*
Amygdala	−0.006	−0.044	0.033	−1.335	0.770
Hippocampus	−0.006	−0.032	0.019	−2.518	0.614
Caudate	−0.010	−0.048	0.028	−6.135	0.602
Putamen	−0.006	−0.038	0.027	−3.676	0.736
Thalamus	−0.030	−0.056	−0.005	−22.209	0.020*
Pallidum	0.009	−0.027	0.046	2.158	0.627

* p < 0.05.

** FDR-BH corrected p < 0.05.

**Table 5:** Prenatal infection trimester 3 and child brain volumes (interaction effect).

	Standardized β-coefficient	95 % Confidence interval		B-coefficient	P-value
Total brain volume	0.030	0.003	0.057	3594.003	0.029*
Banks of the superior temporal sulcus	0.000	−0.029	0.028	−0.178	0.978
Caudal anterior cingulate	0.029	−0.005	0.063	12.696	0.095
Caudal middle frontal	0.045	0.007	0.083	60.455	0.020*
Cuneus	0.014	−0.010	0.038	9.067	0.260
Entorhinal	0.012	−0.027	0.051	5.307	0.543
Fusiform gyrus	0.040	0.006	0.075	57.257	0.021*
Inferior parietal	0.028	−0.004	0.061	68.991	0.090
Inferior temporal	0.040	0.003	0.077	78.391	0.032*
Isthmus cingulate	0.005	−0.016	0.025	2.323	0.646
Lateral occipital	0.014	−0.011	0.039	28.510	0.266
Lateral orbitofrontal	0.064	0.014	0.113	95.609	0.011*
Lingual	−0.013	−0.036	0.009	−15.510	0.236
Medial orbitofrontal	0.036	−0.010	0.083	39.143	0.123
Middle temporal	0.064	0.024	0.104	125.279	0.002**
Parahippocampal	0.029	−0.005	0.064	9.195	0.094
Paracentral	0.036	0.004	0.068	20.055	0.027*
Pars opercularis	0.023	−0.006	0.052	18.385	0.120
Pars orbitalis	0.073	0.025	0.120	42.495	0.003**
Pars triangularis	0.017	−0.017	0.050	13.540	0.327
Pericalcarine	0.015	−0.019	0.049	7.008	0.396
Postcentral	0.026	−0.001	0.053	39.162	0.057
Posterior cingulate	0.017	−0.008	0.041	9.198	0.186
Precentral	0.020	−0.016	0.056	36.078	0.270
Precuneus	0.027	−0.002	0.057	47.859	0.066
Rostral anterior cingulate	0.073	0.025	0.121	48.886	0.003**
Rostral middle frontal	0.043	0.001	0.085	140.353	0.043*
Superior frontal	0.059	0.022	0.096	210.506	0.002**
Superior parietal	0.051	0.012	0.089	117.586	0.009*
Superior temporal	0.031	−0.005	0.067	55.066	0.087
Supramarginal	0.018	−0.012	0.048	38.474	0.234
Frontal pole	0.042	−0.003	0.088	11.987	0.068
Temporal pole	0.076	0.025	0.127	40.457	0.003**
Transverse temporal	0.004	−0.021	0.030	0.853	0.734
Insula	0.027	−0.004	0.058	25.792	0.091
Cerebellum	0.025	−0.011	0.061	164.918	0.177
Amygdala	0.035	−0.007	0.076	7.999	0.099
Hippocampus	0.017	−0.010	0.045	6.714	0.210
Caudate	0.052	0.011	0.092	31.260	0.012*
Putamen	0.022	−0.013	0.057	14.070	0.224
Thalamus	−0.027	−0.055	0.000	−19.898	0.051
Pallidum	0.034	−0.005	0.073	7.987	0.089

* p < 0.05.

** FDR-BH corrected p < 0.05.

## Data Availability

The authors do not have permission to share data. Data from this study are available upon reasonable request to the director of the Generation R Study (generationr@erasmusmc.nl), subject to local, national, and European rules and regulations.
