# Supplementary material for: Examining longitudinal associations between prenatal exposure to infections and child brain morphology
Source: Brain Behav Immun. Author manuscript; Available in PMC 2024 Jul 6. (PMC7616133; doi:10.1016/j.bbi.2024.05.014)
Supplement: Supplementary materials [file EMS196866-supplement-Supplementary_materials.docx]

**Examining longitudinal associations between prenatal exposure to infections and child brain morphology**

Anna Suleri BSc^1,2^, Carolin Gaiser MSc^1,2,3^, Charlotte A.M. Cecil PhD^1,4,5^, Annet Dijkzeul MSc^1,2^, Alexander Neumann PhD^1,2^, Jeremy A. Labrecque PhD^4^, Tonya White MD PhD^6^, Veerle Bergink MD PhD^7,8^*, Ryan L. Muetzel PhD^1,9^

^1^Department of Child and Adolescent Psychiatry/Psychology, Erasmus MC University Medical Center, The Netherlands.

^2^The Generation R Study Group, Erasmus MC University Medical Center, Rotterdam, the Netherlands.

^3^Department of Neuroscience, Erasmus MC University Medical Center, Rotterdam, the Netherlands.

^4^Department of Epidemiology, Erasmus MC University Medical Center, Rotterdam, the Netherlands.

^5^Department of Biomedical Data Sciences, Molecular Epidemiology, Leiden University Medical Center, Leiden, The Netherlands.

^6^Section on Social and Cognitive Developmental Neuroscience, National Institute of Mental Health, Bethesda, Maryland, USA.

^7^Department of Psychiatry, Icahn School of Medicine at Mount Sinai, New York, USA.

^8^Department of Psychiatry, Erasmus MC University Medical Center, Rotterdam, The Netherlands.

^9^Department of Radiology and Nuclear Medicine, Erasmus University Medical Center, Rotterdam, The Netherlands.

*Corresponding author, [veerle.bergink@mssm.edu](mailto:veerle.bergink@mssm.edu)

**SUPPLEMENTARY INFORMATION**

**SUPPLEMENTARY TEXT**

1. **Image quality**

For the imaging wave at mean age 5 and 10 years, we evaluated processed images to assess the quality of segmentation using a six-point Likert scale. The scale ranged from 0 (unusable) to 5 (excellent), with intermediate ratings of 1 to 4 representing decreasing quality. As such, a systematic approach was used to evaluate the quality of the structural MRI scans. This approach involves four key measures: the cerebellum's foliation, the interface between gray and white matter, the presence of ringing artifacts in the image, and the accuracy of subcortical structure segmentation (specifically, caudate and putamen). An image is considered excellent when both anterior and posterior cerebellar foliation are distinct without any blurring. A good quality image exhibits some cerebellar foliation blurring but retains a clear gray/white matter interface. It also shows minimal ringing in both anterior and posterior brain regions across multiple axial sections. Conversely, a poor-quality image displays blurred gray/white matter interfaces, ringing artifacts, and blurring at the borders of subcortical regions. Scans rated as poor or unusable were excluded from further analysis.

For the age group of 13 to 16 years, two independent raters evaluated processed images using a three-point Likert scale: good, questionable, or poor. Images consistently rated as "poor" were not considered. In cases of rater disagreement on data usability, a third rater provided a final assessment, or a consensus was reached through expert discussions. A subset of images was evaluated by a single rater and compared with automated quality assessment scores generated by an in-house support vector machine learning algorithm. The automated quality assessment demonstrated high sensitivity (83%) and moderate specificity (74%). Consequently, scans were deemed to have good quality and included if both the human rater and automated quality assessment indicated so. Scans identified as unusable by the automated quality assessment or those involving rater-automated quality assessment discrepancies underwent assessment by an additional rater to determine their usability.

1. **Nonresponse analysis**

Our nonresponse analysis revealed that mothers included in our study compared to the Generation R baseline cohort were of higher age (mean difference=1.3 years, df=4173, p<2.2e-16), more often of Dutch national background (χ^2^=265, df=1, p<2.2e-16), used more often tobacco prenatally (χ^2^=33.6, df=2, p=5.0e-08), had a lower psychopathology sum score (mean difference= -0.09 GSI, df=4982, p<2.2e-16), more likely to complete a higher education (χ^2^=269, df=2, p<2.2e-16) and had a higher household income (χ^2^=190, df=1, p<2.2e-16). There were no differences in prenatal infection exposure (mean difference= -0.06 sum score, df=4883, p=0.3) nor in SSRI usage (χ^2^=3, df=4, p=0.5). Children of included mothers had fewer behavioral problems (mean difference = -1.56 CBCL score, df=4040, p=5.0e-04) and fewer autistic traits (mean difference= -0.038 SRS total score, df=4007, p=5.13e-08). There was no difference between child’s sex (χ^2^=4, df=1, p=0.05).

1. **Power analysis for interaction effect in mixed-effects model**

We conducted a power analysis to detect the minimum effect size we were able to observe in the interaction models with time, given our sample size. We used the ‘simr’ package in R which conducts a power analysis by employing Monte Carlo simulations to estimate statistical power of a linear mixed-effects model. The simulations (n=1,000) showed that the minimum effect size we were able to detect for the interaction effect between infections and time given our data and model is 0.0042. We additionally used the web-based glimmpse tool for mixed-effects models, to calculate how powered we were to detect our observed effect size, given the correlations in our dataset between each outcome per timepoint. The result of this tool was that we had a power of 0.8.

**SUPPLEMENTARY FIGURES**

**
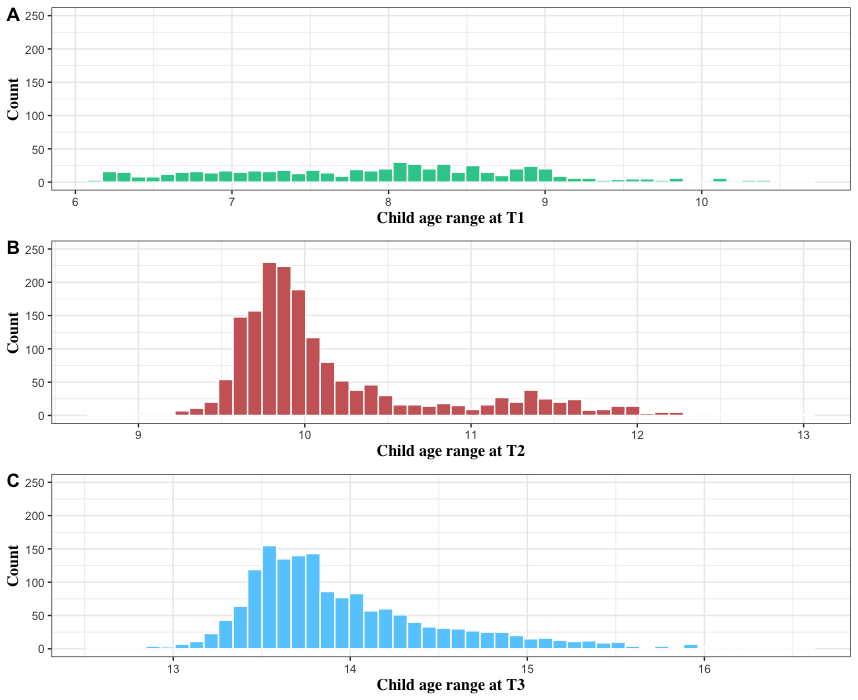
**

**Figure S1.** Histogram depicting age range of the child per timepoint.


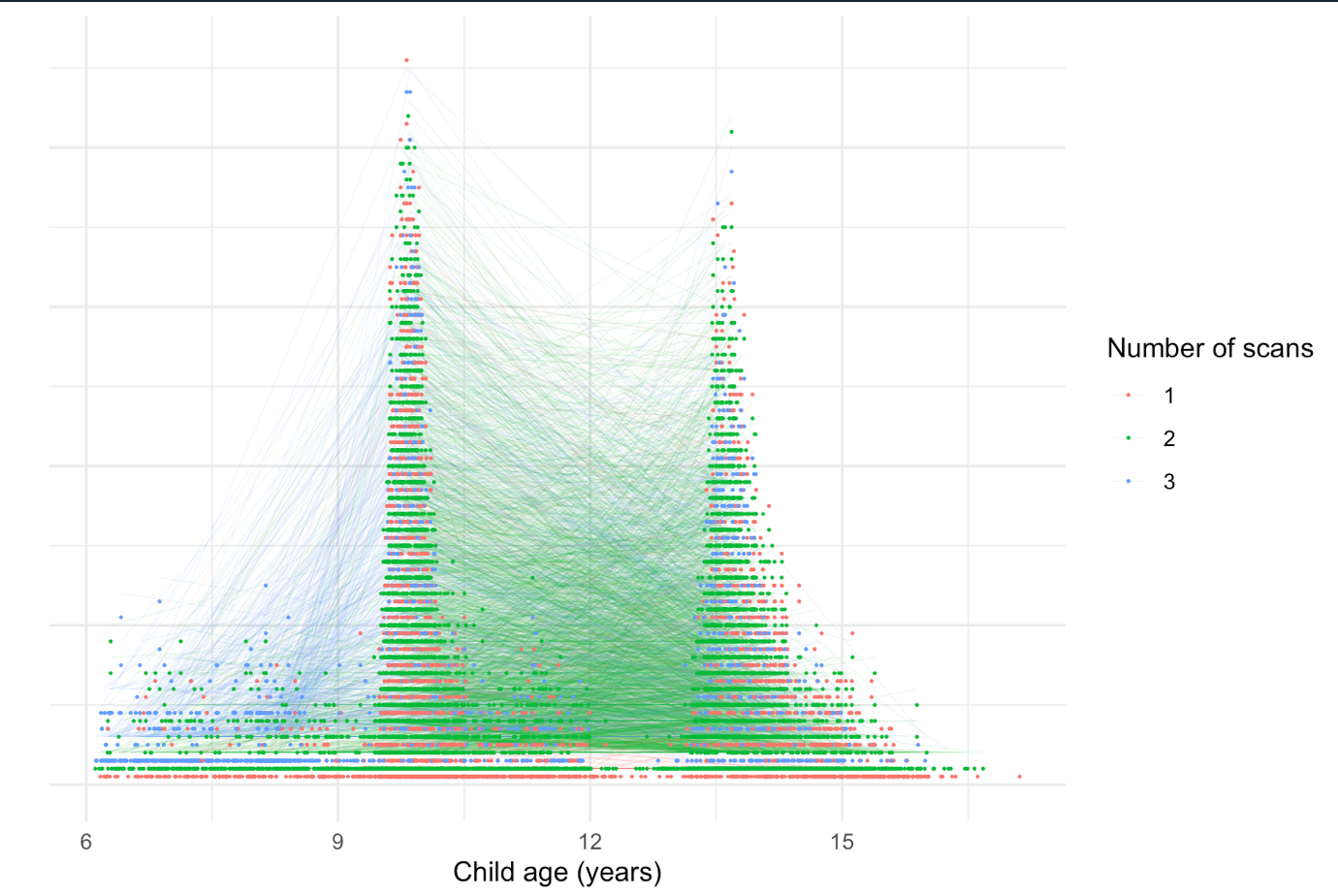


**Figure S2**. Repeated number of scans per subject over time.


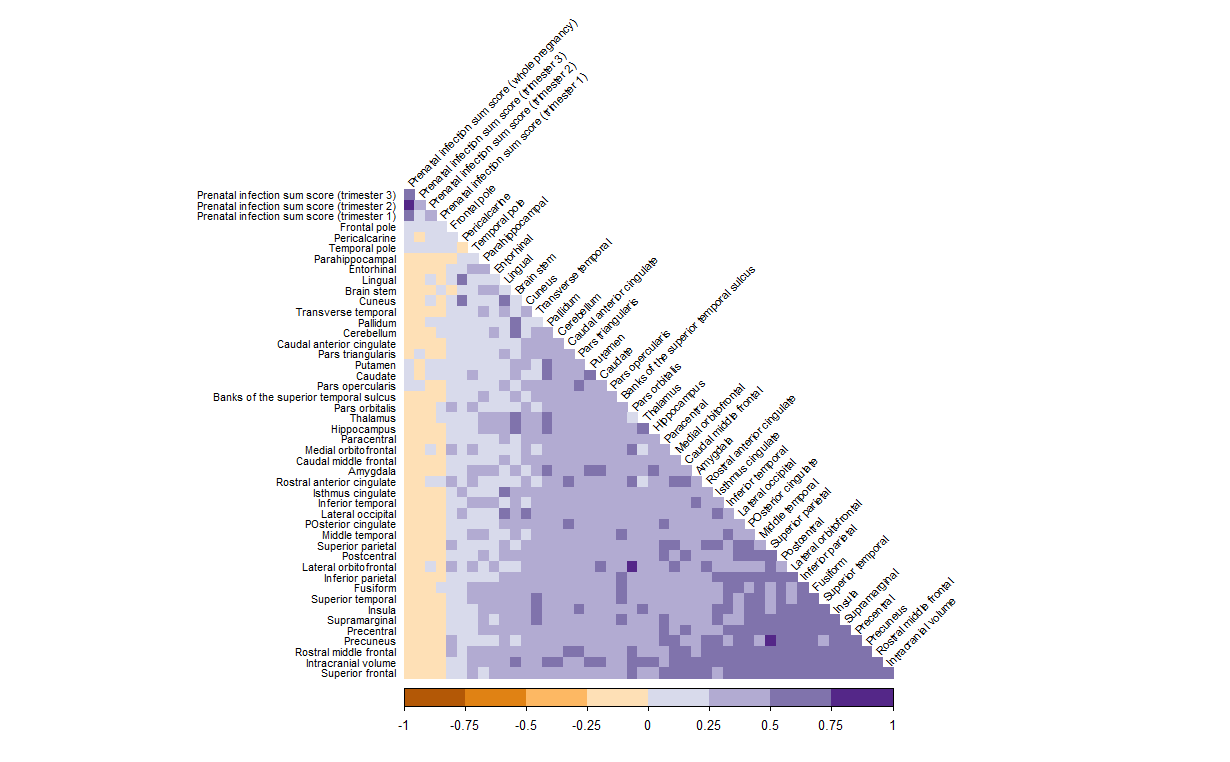


**Figure S3**. Correlation plot.


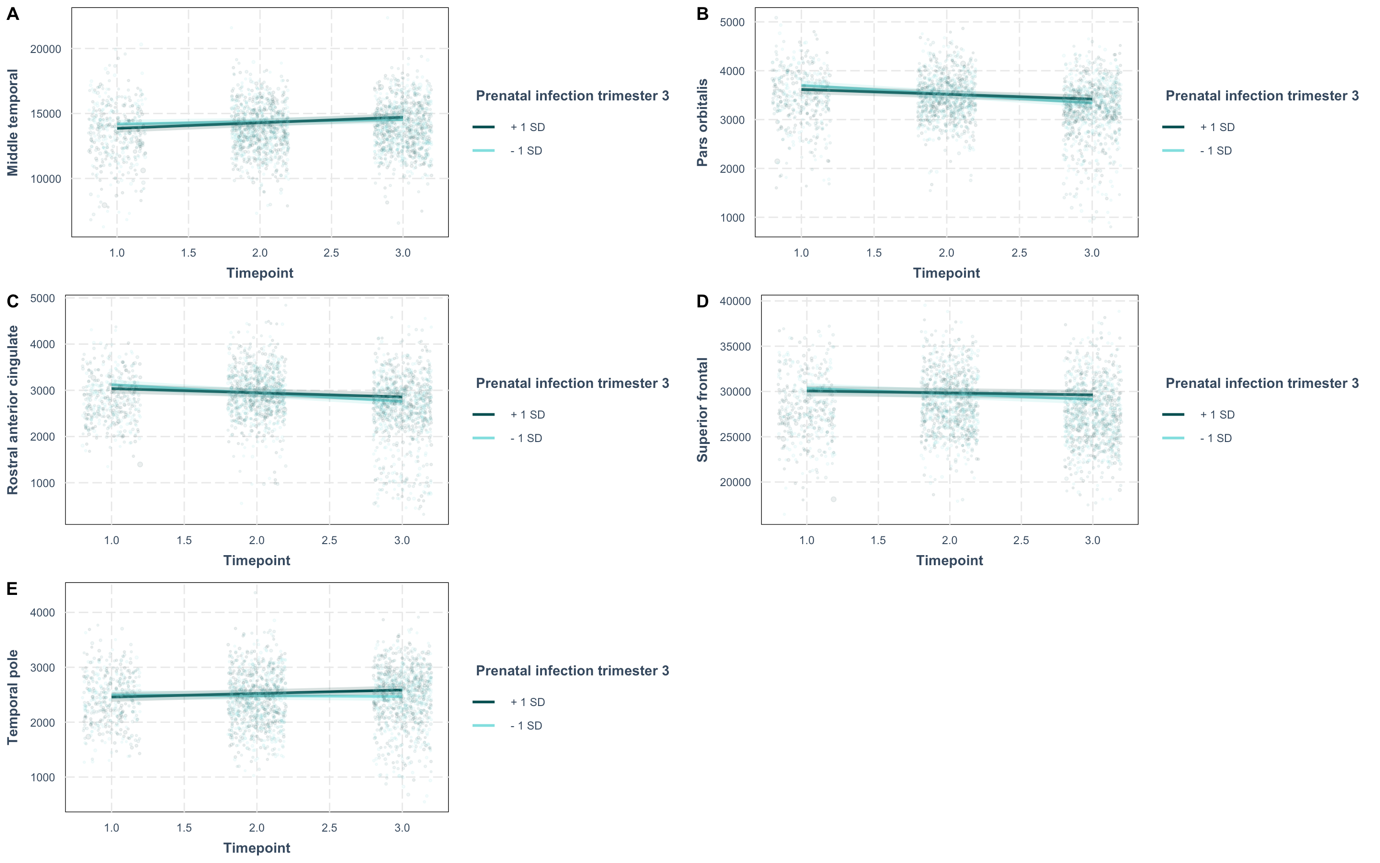


**Figure S4**. Significant brain regions (p_fdr_<0.05) including data points.


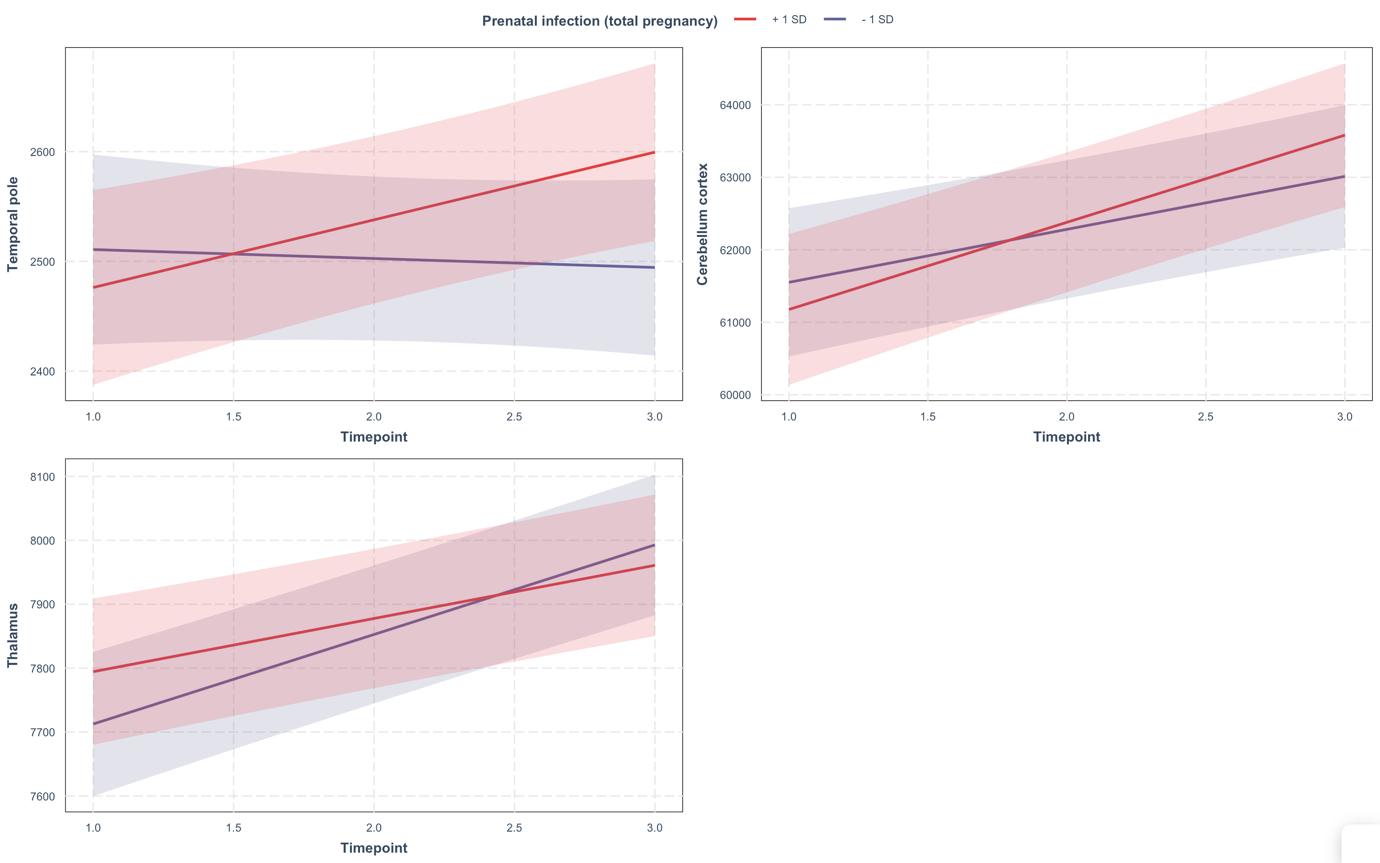


**Figure S5**. Longitudinal plot for infections in total pregnancy for nominal significant results.


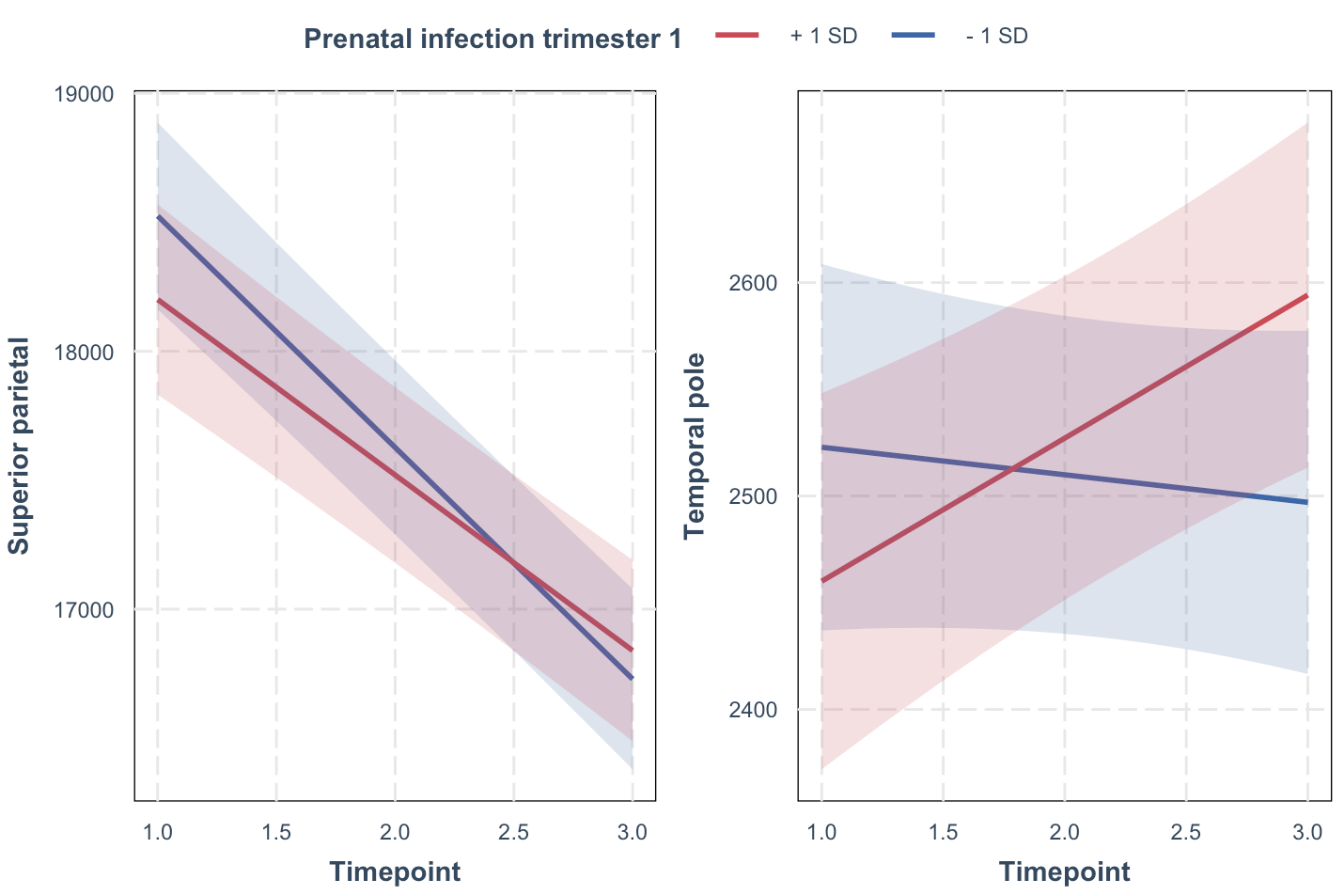


**Figure S6**. Longitudinal plot for trimester 1 infections for nominal significant results.


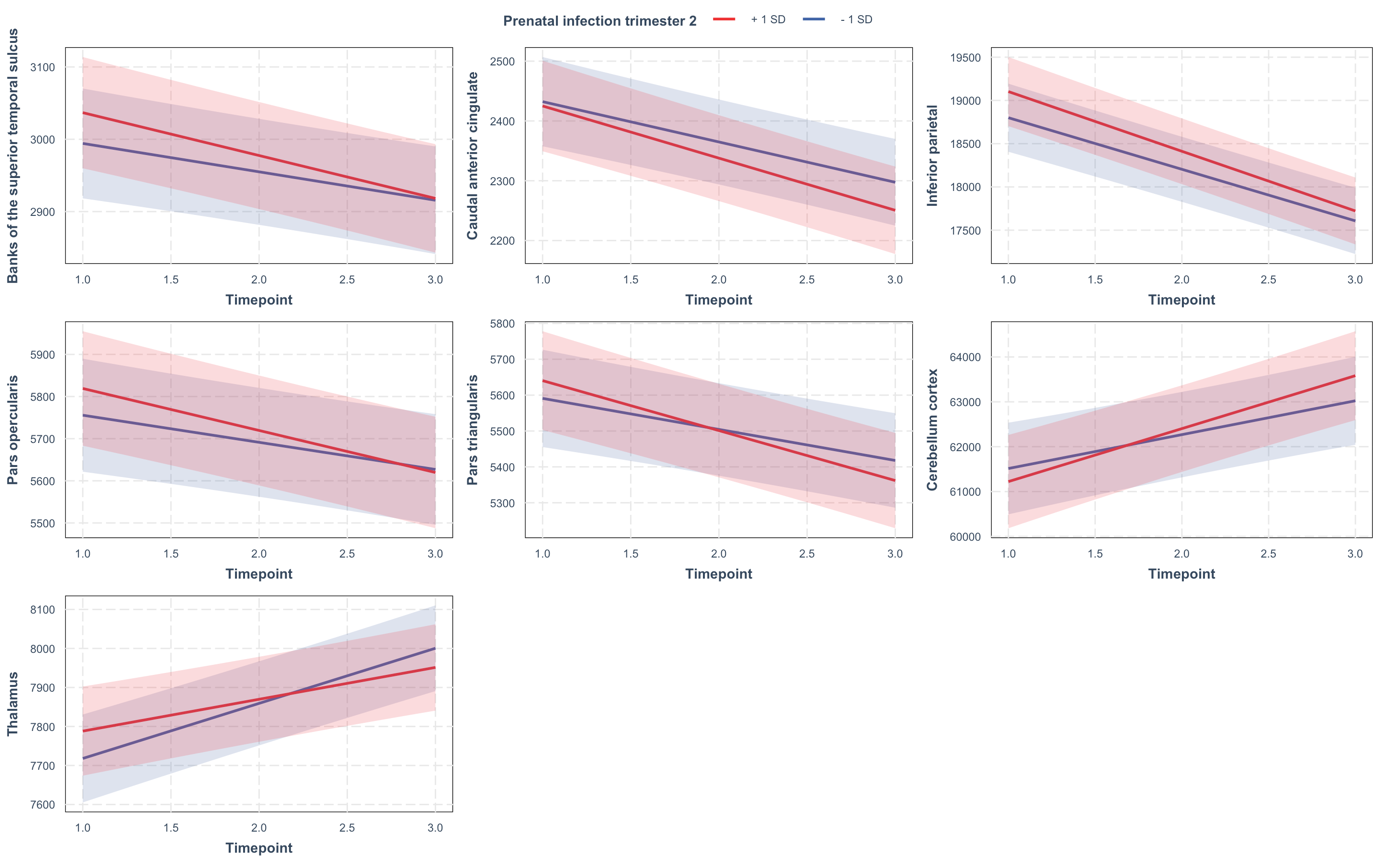


**Figure S7**. Longitudinal plot for trimester 2 infections for nominal significant results.


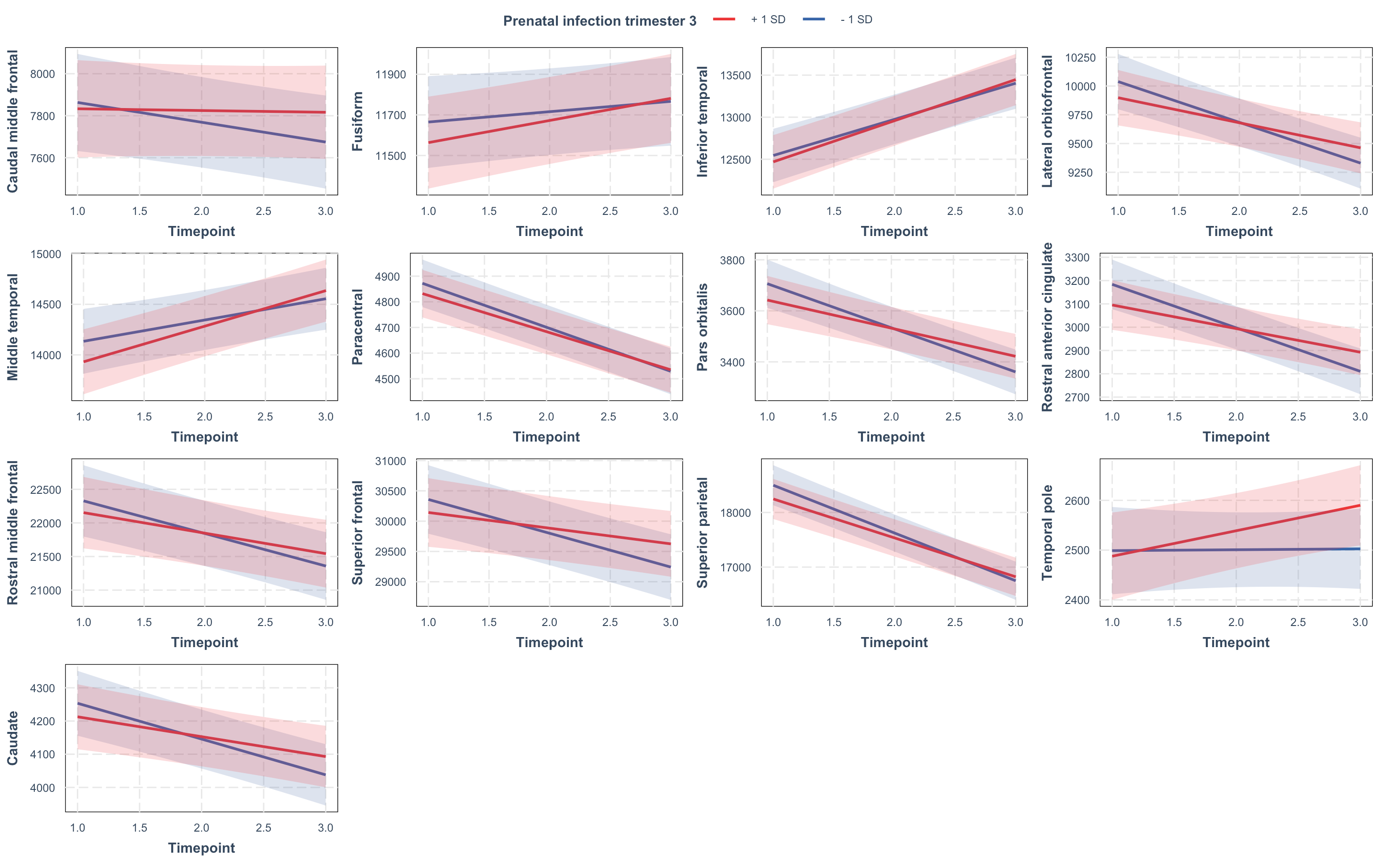


**Figure S8**. Longitudinal plot for trimester 3 infections for nominal significant results.


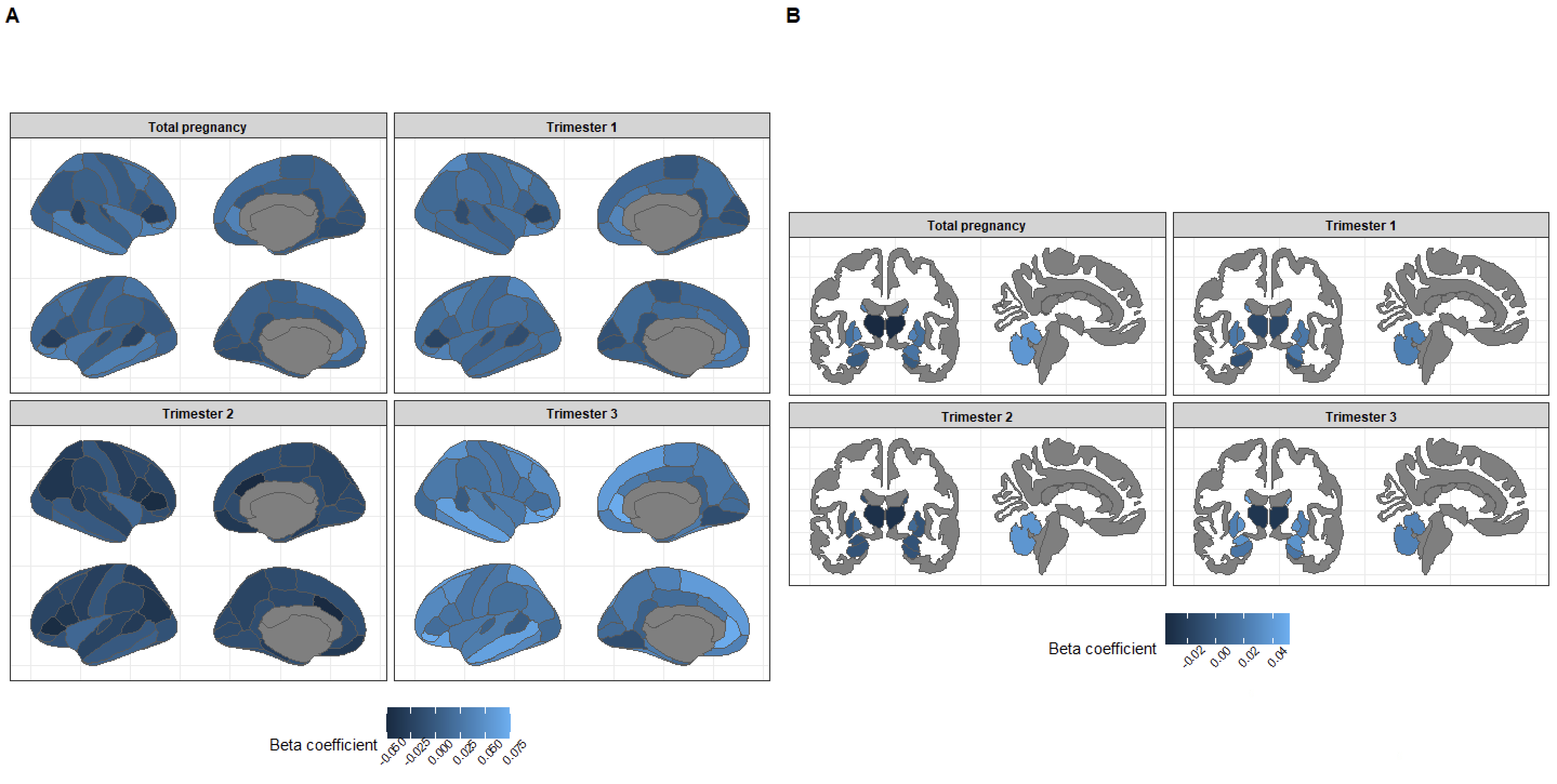


**Figure S9**. Effect estimates of all brain regions for all results of the primary analysis (interaction model, no ICV adjustment).

**SUPPLEMENTARY TABLES**

**Table S1.** Prenatal infection and child brain volumes (main effect)

|  | **Standardized**  $\boldsymbol{\beta}$**-coefficient** | **95% Confidence interval** | | **B-coefficient** | **P-value** |
| --- | --- | --- | --- | --- | --- |
| Total brain volume | -0.020 | -0.074 | 0.033 | -1096.384 | 0.453 |
| Banks of the superior temporal sulcus | -0.012 | -0.066 | 0.042 | -2.403 | 0.666 |
| Caudal anterior cingulate | -0.033 | -0.086 | 0.020 | -6.544 | 0.219 |
| Caudal middle frontal | 0.008 | -0.044 | 0.061 | 5.089 | 0.757 |
| Cuneus | 0.011 | -0.040 | 0.062 | 3.182 | 0.678 |
| Entorhinal | 0.007 | -0.045 | 0.059 | 1.432 | 0.785 |
| Fusiform gyrus | 0.016 | -0.034 | 0.065 | 10.150 | 0.532 |
| Inferior parietal | 0.017 | -0.033 | 0.068 | 18.976 | 0.508 |
| Inferior temporal | 0.001 | -0.049 | 0.050 | 0.506 | 0.982 |
| Isthmus cingulate | 0.027 | -0.022 | 0.076 | 5.965 | 0.275 |
| Lateral occipital | 0.018 | -0.029 | 0.065 | 16.784 | 0.453 |
| Lateral orbitofrontal | 0.015 | -0.030 | 0.060 | 10.259 | 0.509 |
| Lingual | 0.020 | -0.031 | 0.071 | 10.487 | 0.444 |
| Medial orbitofrontal | 0.009 | -0.033 | 0.051 | 4.441 | 0.669 |
| Middle temporal | 0.011 | -0.039 | 0.061 | 9.711 | 0.669 |
| Parahippocampal | -0.021 | -0.074 | 0.032 | -2.961 | 0.439 |
| Paracentral | -0.010 | -0.061 | 0.041 | -2.486 | 0.705 |
| Pars opercularis | 0.031 | -0.021 | 0.083 | 11.339 | 0.247 |
| Pars orbitalis | 0.014 | -0.032 | 0.060 | 3.762 | 0.545 |
| Pars triangularis | 0.014 | -0.038 | 0.065 | 4.986 | 0.606 |
| Pericalcarine | 0.031 | -0.023 | 0.085 | 6.692 | 0.257 |
| Postcentral | -0.002 | -0.052 | 0.049 | -1.140 | 0.947 |
| Posterior cingulate | -0.016 | -0.067 | 0.036 | -3.906 | 0.551 |
| Precentral | -0.001 | -0.051 | 0.048 | -1.002 | 0.960 |
| Precuneus | -0.011 | -0.059 | 0.036 | -8.774 | 0.646 |
| Rostral anterior cingulate | 0.031 | -0.014 | 0.076 | 9.401 | 0.177 |
| Rostral middle frontal | 0.005 | -0.044 | 0.054 | 7.309 | 0.842 |
| Superior frontal | 0.019 | -0.029 | 0.067 | 30.935 | 0.435 |
| Superior parietal | -0.017 | -0.065 | 0.031 | -17.734 | 0.488 |
| Superior temporal | -0.002 | -0.054 | 0.051 | -1.405 | 0.947 |
| Supramarginal | 0.011 | -0.038 | 0.060 | 10.858 | 0.649 |
| Frontal pole | 0.020 | -0.023 | 0.062 | 2.537 | 0.359 |
| Temporal pole | 0.057 | 0.011 | 0.104 | 13.871 | 0.014* |
| Transverse temporal | -0.061 | -0.115 | -0.007 | -5.386 | 0.025* |
| Insula | 0.004 | -0.046 | 0.053 | 1.644 | 0.882 |
| Cerebellum | 0.013 | -0.035 | 0.060 | 37.913 | 0.604 |
| Amygdala | 0.022 | -0.025 | 0.069 | 2.341 | 0.351 |
| Hippocampus | 0.018 | -0.032 | 0.068 | 3.161 | 0.478 |
| Caudate | 0.042 | -0.007 | 0.090 | 11.494 | 0.091 |
| Putamen | 0.023 | -0.025 | 0.071 | 6.717 | 0.349 |
| Thalamus | 0.012 | -0.037 | 0.061 | 3.955 | 0.628 |
| Pallidum | 0.009 | -0.040 | 0.058 | 0.951 | 0.724 |
| *p<0.05  **FDR-BH corrected p<0.05 | | | | | |

**Table S2.** Prenatal infection and child brain volumes (interaction effect, ICV-adjusted)

|  | **Standardized**  $\boldsymbol{\beta}$**-coefficient** | **95% Confidence interval** | | **B-coefficient** | **P-value** |
| --- | --- | --- | --- | --- | --- |
| Banks of the superior temporal sulcus | -0.035 | -0.064 | -0.006 | -7.045 | 0.018* |
| Caudal anterior cingulate | -0.027 | -0.057 | 0.004 | -5.279 | 0.089 |
| Caudal middle frontal | -0.006 | -0.038 | 0.026 | -3.554 | 0.722 |
| Cuneus | -0.015 | -0.039 | 0.010 | -4.330 | 0.236 |
| Entorhinal | -0.006 | -0.045 | 0.033 | -1.266 | 0.749 |
| Fusiform gyrus | -0.002 | -0.035 | 0.032 | -1.021 | 0.925 |
| Inferior parietal | -0.026 | -0.056 | 0.005 | -28.641 | 0.102 |
| Inferior temporal | 0.000 | -0.035 | 0.035 | -0.002 | 0.999 |
| Isthmus cingulate | -0.023 | -0.043 | -0.004 | -5.122 | 0.019* |
| Lateral occipital | -0.004 | -0.029 | 0.020 | -4.093 | 0.726 |
| Lateral orbitofrontal | 0.000 | -0.039 | 0.038 | -0.209 | 0.987 |
| Lingual | -0.012 | -0.034 | 0.010 | -6.371 | 0.281 |
| Medial orbitofrontal | -0.026 | -0.063 | 0.011 | -12.785 | 0.164 |
| Middle temporal | 0.009 | -0.028 | 0.046 | 7.906 | 0.637 |
| Parahippocampal | -0.007 | -0.041 | 0.027 | -0.970 | 0.695 |
| Paracentral | -0.018 | -0.048 | 0.012 | -4.525 | 0.234 |
| Pars opercularis | -0.020 | -0.048 | 0.008 | -7.284 | 0.165 |
| Pars orbitalis | 0.002 | -0.038 | 0.042 | 0.483 | 0.928 |
| Pars triangularis | -0.039 | -0.072 | -0.005 | -14.187 | 0.023* |
| Pericalcarine | -0.006 | -0.040 | 0.027 | -1.388 | 0.706 |
| Postcentral | -0.006 | -0.033 | 0.021 | -4.044 | 0.660 |
| Posterior cingulate | -0.013 | -0.037 | 0.011 | -3.169 | 0.304 |
| Precentral | -0.028 | -0.059 | 0.004 | -22.384 | 0.085 |
| Precuneus | -0.017 | -0.042 | 0.009 | -13.094 | 0.210 |
| Rostral anterior cingulate | 0.002 | -0.034 | 0.037 | 0.475 | 0.931 |
| Rostral middle frontal | -0.017 | -0.054 | 0.020 | -24.943 | 0.375 |
| Superior frontal | -0.015 | -0.044 | 0.014 | -24.181 | 0.317 |
| Superior parietal | -0.002 | -0.035 | 0.032 | -1.859 | 0.917 |
| Superior temporal | -0.014 | -0.050 | 0.022 | -11.114 | 0.444 |
| Supramarginal | -0.018 | -0.046 | 0.010 | -17.283 | 0.199 |
| Frontal pole | 0.013 | -0.031 | 0.057 | 1.672 | 0.558 |
| Temporal pole | 0.041 | -0.006 | 0.088 | 9.947 | 0.085 |
| Transverse temporal | -0.013 | -0.038 | 0.012 | -1.153 | 0.307 |
| Insula | 0.006 | -0.026 | 0.038 | 2.629 | 0.711 |
| Cerebellum | 0.017 | -0.016 | 0.050 | 51.145 | 0.311 |
| Amygdala | -0.013 | -0.047 | 0.021 | -1.329 | 0.466 |
| Hippocampus | -0.023 | -0.048 | 0.001 | -4.108 | 0.060 |
| Caudate | -0.008 | -0.038 | 0.023 | -2.099 | 0.626 |
| Putamen | -0.012 | -0.042 | 0.019 | -3.430 | 0.451 |
| Thalamus | -0.046 | -0.075 | -0.017 | -15.114 | 0.002* |
| Pallidum | 0.001 | -0.034 | 0.036 | 0.129 | 0.945 |
| *p<0.05  **FDR-BH corrected p<0.05 | | | | | |

**Table S3.** Prenatal infection in trimester 3 and FDR-significant brain regions (in interaction model) after mutually adjusting for infections in trimester 1 and 2 and their interaction with time.

|  | **Standardized**  $\boldsymbol{\beta}$**-coefficient** | **95% Confidence interval** | | **P-value** |
| --- | --- | --- | --- | --- |
| Middle temporal | 0.045 | 0.004 | 0.086 | 0.032** |
| Pars orbitalis | 0.059 | 0.008 | 0.110 | 0.023** |
| Rostral anterior cingulate | 0.069 | 0.017 | 0.120 | 0.009** |
| Superior frontal | 0.047 | 0.008 | 0.085 | 0.018** |
| Temporal pole | 0.045 | -0.010 | 0.099 | 0.110 |
| *p<0.05  **FDR-BH corrected p<0.05 | | | | |

**Table S4**. Complete case analysis for each trimester and FDR-significant brain regions.

|  | **Beta** | **SE** | **P value** |
| --- | --- | --- | --- |
| *Trimester 1* | | | |
| Middle temporal | 0.033 | 0.024 | 0.178 |
| Pars orbitalis | 0.037 | 0.035 | 0.288 |
| Rostral anterior cingulate cortex | 0.061 | 0.035 | 0.091 |
| Superior frontal | 0.033 | 0.024 | 0.166 |
| Temporal pole | 0.094 | 0.040 | 0.018* |
| *Trimester 2* | | | |
| Middle temporal | 0.002 | 0.024 | 0.919 |
| Pars orbitalis | 0.033 | 0.034 | 0.342 |
| Rostral anterior cingulate cortex | 0.073 | 0.035 | 0.040* |
| Superior frontal | 0.020 | 0.023 | 0.382 |
| Temporal pole | 0.067 | 0.039 | 0.092 |
| *Trimester 3* | | | |
| Middle temporal | 0.021 | 0.024 | 0.384 |
| Pars orbitalis | 0.063 | 0.035 | 0.071 |
| Rostral anterior cingulate cortex | 0.105 | 0.036 | 0.003* |
| Superior frontal | 0.063 | 0.023 | 0.007* |
| Temporal pole | 0.098 | 0.040 | 0.014* |
| *p<0.05  Of note, 530 participants had 1 scan, 1374 participants had 2 scans, and 251 participants had 3 scans. | | | |

**Table S5**. Interaction model between maternal education (as proxy for SES) and time and changes in five FDR-significant brain regions

|  | **Standardized**  $\boldsymbol{\beta}$**-coefficient** | **95% Confidence interval** | | **P-value** |
| --- | --- | --- | --- | --- |
| Middle temporal | -0.056 | -0.133 | 0.021 | 0.155 |
| Pars orbitalis | -0.059 | -0.155 | 0.037 | 0.229 |
| Rostral anterior cingulate | -0.035 | -0.132 | 0.063 | 0.485 |
| Superior frontal | -0.048 | -0.121 | 0.025 | 0.199 |
| Temporal pole | 0.001 | -0.103 | 0.105 | 0.990 |
| *p<0.05  **FDR-BH corrected p<0.05 | | | | |

**Table S6**. Moderation effect of environmental factors on the association between prenatal infections and FDR-significant brain regions (interaction model with time)

|  | **Standardized**  $\boldsymbol{\beta}$**-coefficient** | **95% Confidence interval** | | | **P-value** |
| --- | --- | --- | --- | --- | --- |
| Moderator: maternal education | | | | | |
| Middle temporal | 0.060 | -0.121 | 0.242 | | 0.514 |
| Pars orbitalis | 0.019 | -0.209 | 0.246 | | 0.873 |
| Rostral anterior cingulate | -0.032 | -0.261 | 0.198 | | 0.788 |
| Superior frontal | -0.039 | -0.211 | 0.134 | | 0.660 |
| Temporal pole | -0.015 | -0.263 | 0.233 | | 0.904 |
| Moderator: maternal psychopathology | | | | | |
| Middle temporal | 0.014 | -0.091 | | 0.121 | 0.781 |
| Pars orbitalis | -0.024 | -0.155 | | 0.106 | 0.710 |
| Rostral anterior cingulate | -0.069 | -0.203 | | 0.065 | 0.312 |
| Superior frontal | -0.043 | -0.141 | | 0.055 | 0.393 |
| Temporal pole | 0.070 | -0.075 | | 0.217 | 0.341 |
| Moderator: maternal alcohol use | | | | | |
| Middle temporal | 0.162 | -0.265 | | 0.591 | 0.455 |
| Pars orbitalis | 0.222 | -0.288 | | 0.733 | 0.393 |
| Rostral anterior cingulate | 0.251 | -0.261 | | 0.764 | 0.336 |
| Superior frontal | 0.395 | -0.012 | | 0.804 | 0.057 |
| Temporal pole | 0.204 | -0.343 | | 0.752 | 0.463 |
| Moderator: postnatal life events | | | | | |
| Middle temporal | 0.105 | -0.159 | | 0.369 | 0.437 |
| Pars orbitalis | 0.134 | -0.198 | | 0.466 | 0.430 |
| Rostral anterior cingulate | 0.076 | -0.258 | | 0.410 | 0.656 |
| Superior frontal | 0.063 | -0.186 | | 0.312 | 0.618 |
| Temporal pole | 0.213 | -0.147 | | 0.573 | 0.246 |
| Moderator: postnatal direct victimization | | | | | |
| Middle temporal | -0.070 | -0.336 | | 0.194 | 0.600 |
| Pars orbitalis | -0.032 | -0.366 | | 0.302 | 0.850 |
| Rostral anterior cingulate | -0.288 | -0.62 | | 0.047 | 0.092 |
| Superior frontal | -0.197 | -0.447 | | 0.052 | 0.121 |
| Temporal pole | 0.065 | -0.298 | | 0.429 | 0.724 |
| *p<0.05  **FDR-BH corrected p<0.05 | | | | | |

**Table S7**. Sensitivity analysis additionally adjusting for immune status mother in interaction model for significant brain regions.

|  | **Standardized**  $\boldsymbol{\beta}$**-coefficient** | **95% Confidence interval** | | **P-value** |
| --- | --- | --- | --- | --- |
| Middle temporal | 0.050 | 0.010 | 0.089 | 0.015 |
| Pars orbitalis | 0.075 | 0.026 | 0.124 | 0.003 |
| Rostral anterior cingulate | 0.083 | 0.033 | 0.132 | 0.001 |
| Superior frontal | 0.055 | 0.018 | 0.092 | 0.003 |
| Temporal pole | 0.064 | 0.011 | 0.117 | 0.018 |
| *p<0.05  **FDR-BH corrected p<0.05 | | | | |

**Table S8**. Association between infection sum score without fever on FDR-significant findings

|  | **Standardized**  $\boldsymbol{\beta}$**-coefficient** | **95% Confidence interval** | | **P-value** |
| --- | --- | --- | --- | --- |
| Middle temporal | 0.036 | 0.003 | 0.070 | 0.032** |
| Pars orbitalis | 0.021 | -0.020 | 0.063 | 0.313 |
| Rostral anterior cingulate | 0.048 | 0.006 | 0.090 | 0.024** |
| Superior frontal | 0.035 | 0.003 | 0.066 | 0.031** |
| Temporal pole | 0.066 | 0.020 | 0.111 | 0.005** |
| *p<0.05  **FDR-BH corrected p<0.05 | | | | |

**Table S9**. Interaction between infection sum score without fever and fever on FDR-significant findings

|  | **Standardized**  $\boldsymbol{\beta}$**-coefficient** | **95% Confidence interval** | | **P-value** |
| --- | --- | --- | --- | --- |
| Middle temporal | -0.003 | -0.138 | 0.132 | 0.965 |
| Pars orbitalis | 0.106 | -0.066 | 0.279 | 0.228 |
| Rostral anterior cingulate | 0.056 | -0.117 | 0.229 | 0.523 |
| Superior frontal | -0.026 | -0.155 | 0.102 | 0.687 |
| Temporal pole | -0.017 | -0.206 | 0.172 | 0.862 |
| *p<0.05  **FDR-BH corrected p<0.05 | | | | |

**Table S10.** Prenatal infection in trimester 3 and FDR-significant brain regions (in interaction model) after adjusting for child age instead of time of visit.

|  | **Standardized**  $\boldsymbol{\beta}$**-coefficient** | **95% Confidence interval** | | **P-value** |
| --- | --- | --- | --- | --- |
| Middle temporal | 0.012 | 0.000 | 0.023 | 0.047* |
| Pars orbitalis | 0.015 | 0.001 | 0.029 | 0.038* |
| Rostral anterior cingulate | 0.016 | 0.002 | 0.030 | 0.029* |
| Superior frontal | 0.011 | 0.000 | 0.022 | 0.044* |
| Temporal pole | 0.015 | -0.001 | 0.030 | 0.067 |
| *p<0.05 | | | | |

**Table S11**. Comparing model fit between linear and non-linear mixed-effects models (using splines) for the models of prenatal infection in trimester 3 and FDR-significant brain regions (interaction) model.

|  | **AIC** | | | **BIC** | | |
| --- | --- | --- | --- | --- | --- | --- |
|  | Linear model | Non-linear model | Percentage change | Linear model | Non-linear model | Percentage change |
| Middle temporal | 5852.1 | 5900 | 1.3% | 5932.4 | 5980.3 | 0.8% |
| Pars orbitalis | 6245.2 | 6310.9 | 1.1% | 6325.6 | 6391.2 | 1.1% |
| Rostral anterior cingulate | 6220.3 | 6300.6 | 1.3% | 6263.7 | 6344.1 | 1.3% |
| Superior frontal | 5634.8 | 5656.3 | 0.4% | 5715.1 | 5736.6 | 0.4% |
| Temporal pole | 6467.3 | 6471.9 | 0.1% | 6547.6 | 6552.3 | 0.1% |
| AIC = Akaike information criterion; BIC = Bayesian information criterion | | | | | | |

**Table S12**. Prenatal infection in trimester 3 and FDR-significant brain regions (in interaction model) using splines.

|  |  | **Standardized**  $\boldsymbol{\beta}$**-coefficient** | **95% Confidence interval** | | **P-value** |
| --- | --- | --- | --- | --- | --- |
| Middle temporal |  |  |  |  |  |
|  | Knot 1 | 0.028 | -0.045 | 0.613 | 0.091 |
|  | Knot 2 | -0.013 | -0.430 | 0.404 | 0.952 |
|  | Knot 3 | 0.351 | -0.474 | 1.175 | 0.404 |
| Pars orbitalis |  |  |  |  |  |
|  | Knot 1 | -0.126 | -0.467 | 0.214 | 0.467 |
|  | Knot 2 | 0.405 | -0.073 | 0.883 | 0.096 |
|  | Knot 3 | -0.201 | -1.128 | 0.726 | 0.670 |
| Rostral anterior cingulate |  |  |  |  |  |
|  | Knot 1 | -0.130 | -0.464 | 0.205 | 0.447 |
|  | Knot 2 | 0.460 | -0.013 | 0.934 | 0.057 |
|  | Knot 3 | -0.148 | -1.066 | 0.769 | 0.751 |
| Superior frontal |  |  |  |  |  |
|  | Knot 1 | -0.115 | -0.428 | 0.198 | 0.472 |
|  | Knot 2 | 0.455 | 0.061 | 0.848 | 0.024* |
|  | Knot 3 | 0.248 | -0.530 | 1.027 | 0.531 |
| Temporal pole |  |  |  |  |  |
|  | Knot 1 | 0.141 | -0.205 | 0.488 | 0.424 |
|  | Knot 2 | 0.422 | -0.074 | 0.918 | 0.095 |
|  | Knot 3 | 0.693 | -0.264 | 1.651 | 0.155 |
| *p<0.05  **FDR-BH corrected p<0.05 | | | | | |

**Table S13**. Moderation analysis with child sex in the association between prenatal infections and child brain morphology (three-way interaction model)

|  | **Standardized**  $\boldsymbol{\beta}$**-coefficient** | **95% Confidence interval** | | **P-value** |
| --- | --- | --- | --- | --- |
| Total brain volume | -0.014 | -0.066 | 0.037 | 0.584 |
| Banks of the superior temporal sulcus | -0.050 | -0.119 | 0.017 | 0.142 |
| Caudal anterior cingulate | 0.052 | -0.028 | 0.132 | 0.203 |
| Caudal middle frontal | 0.028 | -0.025 | 0.083 | 0.301 |
| Cuneus | 0.034 | -0.044 | 0.112 | 0.396 |
| Entorhinal | 0.048 | -0.021 | 0.119 | 0.174 |
| Fusiform gyrus | 0.045 | -0.009 | 0.101 | 0.103 |
| Inferior parietal | 0.043 | -0.034 | 0.121 | 0.272 |
| Inferior temporal | 0.003 | -0.051 | 0.058 | 0.893 |
| Isthmus cingulate | 0.041 | -0.022 | 0.105 | 0.205 |
| Lateral occipital | 0.018 | -0.054 | 0.091 | 0.616 |
| Lateral orbitofrontal | 0.023 | -0.024 | 0.071 | 0.337 |
| Lingual | -0.053 | -0.129 | 0.023 | 0.172 |
| Medial orbitofrontal | -0.023 | -0.086 | 0.039 | 0.467 |
| Middle temporal | 0.007 | -0.051 | 0.065 | 0.815 |
| Parahippocampal | -0.031 | -0.100 | 0.038 | 0.381 |
| Paracentral | 0.008 | -0.031 | 0.049 | 0.667 |
| Pars opercularis | 0.002 | -0.044 | 0.048 | 0.930 |
| Pars orbitalis | 0.031 | -0.063 | 0.126 | 0.512 |
| Pars triangularis | 0.000 | -0.044 | 0.044 | 0.993 |
| Pericalcarine | 0.004 | -0.082 | 0.092 | 0.915 |
| Postcentral | -0.034 | -0.108 | 0.039 | 0.358 |
| Posterior cingulate | -0.015 | -0.080 | 0.050 | 0.652 |
| Precentral | 0.037 | -0.024 | 0.100 | 0.234 |
| Precuneus | 0.031 | -0.023 | 0.087 | 0.259 |
| Rostral anterior cingulate | 0.041 | -0.050 | 0.133 | 0.372 |
| Rostral middle frontal | 0.026 | -0.038 | 0.090 | 0.425 |
| Superior frontal | -0.010 | -0.078 | 0.058 | 0.774 |
| Superior parietal | -0.012 | -0.061 | 0.037 | 0.633 |
| Superior temporal | 0.013 | -0.032 | 0.059 | 0.564 |
| Supramarginal | 0.018 | -0.049 | 0.087 | 0.590 |
| Frontal pole | 0.012 | -0.044 | 0.068 | 0.674 |
| Temporal pole | 0.048 | -0.044 | 0.140 | 0.307 |
| Transverse temporal | -0.012 | -0.090 | 0.066 | 0.758 |
| Insula | 0.005 | -0.063 | 0.075 | 0.867 |
| Cerebellum | 0.032 | -0.039 | 0.103 | 0.376 |
| Amygdala | -0.021 | -0.088 | 0.046 | 0.541 |
| Hippocampus | 0.000 | -0.057 | 0.057 | 0.998 |
| Caudate | -0.007 | -0.097 | 0.083 | 0.876 |
| Putamen | -0.094 | -0.193 | 0.004 | 0.060 |
| Thalamus | -0.003 | -0.053 | 0.046 | 0.882 |
| Pallidum | -0.027 | -0.088 | 0.034 | 0.384 |
| *p<0.05  **FDR-BH corrected p<0.05 | | | | |

**Table S14**. Prenatal infections in trimester 3 and FDR-significant brain regions (only using neuroimaging wave 2 and 3 data)

|  | **Standardized**  $\boldsymbol{\beta}$**-coefficient** | **95% Confidence interval** | | **P-value** |
| --- | --- | --- | --- | --- |
| Middle temporal | -0.006 | -0.054 | 0.042 | 0.811 |
| Pars orbitalis | 0.036 | -0.035 | 0.106 | 0.323 |
| Rostral anterior cingulate | 0.056 | -0.020 | 0.133 | 0.160 |
| Superior frontal | 0.026 | -0.025 | 0.077 | 0.314 |
| Temporal pole | 0.068 | -0.013 | 0.148 | 0.098 |
| *p<0.05  **FDR-BH corrected p<0.05 | | | | |

**Table S15**. Linear regression models for each time point individually and the significant brain regions

|  | **Standardized**  $\boldsymbol{\beta}$**-coefficient** | **95% Confidence interval** | | **P-value** |
| --- | --- | --- | --- | --- |
| Neuroimaging visit 1 (child mean age 8, n = 582) | | | | |
| Middle temporal | -0.096 | -0.214 | 0.023 | 0.113 |
| Pars orbitalis | -0.067 | -0.172 | 0.038 | 0.212 |
| Rostral anterior cingulate | -0.074 | -0.155 | 0.006 | 0.071 |
| Superior frontal | -0.025 | -0.128 | 0.079 | 0.638 |
| Temporal pole | -0.003 | -0.096 | 0.091 | 0.957 |
| Neuroimaging visit 2 (child mean age 10, n = 1705) | | | | |
| Middle temporal | -0.005 | -0.065 | 0.056 | 0.882 |
| Pars orbitalis | 0.030 | -0.022 | 0.082 | 0.261 |
| Rostral anterior cingulate | 0.007 | -0.045 | 0.059 | 0.793 |
| Superior frontal | 0.043 | -0.015 | 0.101 | 0.146 |
| Temporal pole | 0.033 | -0.031 | 0.096 | 0.312 |
| Neuroimaging visit 3 (child mean age 14, n = 1543) | | | | |
| Middle temporal | 0.018 | -0.038 | 0.075 | 0.529 |
| Pars orbitalis | 0.041 | -0.027 | 0.109 | 0.240 |
| Rostral anterior cingulate | 0.046 | -0.025 | 0.118 | 0.204 |
| Superior frontal | 0.046 | -0.014 | 0.105 | 0.130 |
| Temporal pole | 0.069 | 0.002 | 0.137 | 0.043* |
| *p<0.05  **FDR-BH corrected p<0.05 | | | | |
